# The Intervention Selection Toolbox to improve patient-relevant outcomes: an implementation and qualitative evaluation study in colorectal cancer surgery

**DOI:** 10.1186/s12913-023-09264-3

**Published:** 2023-04-06

**Authors:** Bo Smalbroek, Joanna Vijverberg, Milad Fahim, Lea Dijksman, Douwe Biesma, Anke Smits, Frits van Merode, Paul van der Nat

**Affiliations:** 1 Department of Value-Based Healthcare, St. Antonius Hospital, Nieuwegein, The Netherlands; 2 Department of Surgery, St. Antonius Hospital, Nieuwegein, The Netherlands; 3 Care and Public Health Research Institute (CAPHRI), Maastricht University Medical Centre+, Maastricht, The Netherlands; 4 Department of Internal Medicine, Leiden University Medical Centre, Leiden, Netherlands; 5 Radboud Institute for Health Sciences, Scientific Center for Quality of Healthcare (IQ Healthcare), Radboud University Medical Center, Nijmegen, The Netherlands

**Keywords:** Value-based healthcare, Colorectal, Improvement interventions, Intervention Selection Toolbox, Decision-making, Quality improvement, Healthcare

## Abstract

**Background:**

The concept of value-based healthcare is being used worldwide to improve healthcare. The Intervention Selection Toolbox was developed to bridge the gap of value-based healthcare, between insights in outcomes and actual quality improvement initiatives. In this study we aimed to evaluate the use of the Intervention Selection Toolbox in daily practice of a quality improvement team in a hospital setting.

**Methods:**

A methodological triangulation design was used. The Intervention Selection Toolbox was used by a multidisciplinary quality improvement team for colorectal cancer care in a large teaching hospital. In-depth semi-structured interviews, focusing on the key elements of process evaluation, were conducted after implementation with representatives of the quality improvement team to evaluate the use of the Intervention Selection Toolbox. Quantitative data regarding improvement initiatives and degree of implementation was also collected.

**Results:**

The use of the Intervention Selection Toolbox initially resulted in 80 potential quality improvement initiatives. Eventually, two high potential improvement initiatives were selected. Some components of the toolbox were successfully implemented in daily practice, although ‘standard monitoring’ and ‘causal chain analysis’ proved more difficult to implement. Qualitative analysis was performed with ten members of the multidisciplinary team before thematic saturation occurred. Interviewed members had a wide range in characteristics: age 28–61 years, clinical experience 6–38 years and educational attainment from vocational program to academic doctorate. The Interviews showed added value in the use of the toolbox, but identified time and organizational management as restricting factors.

**Conclusions:**

The Intervention Selection Toolbox is useful to systematically identify improvement initiatives with impact on health outcomes that matter to patients. However, before implementation organizational structure should be optimized to maximize success and efficiency on integration of the Intervention Selection Toolbox.

**Supplementary Information:**

The online version contains supplementary material available at 10.1186/s12913-023-09264-3.

## Background

The need to continuously improve healthcare outcomes has been widely recognized [1–4]. At the same time, managing rising healthcare expenditure in combination with an aging population with increased healthcare utilization has put healthcare systems and governments in the western world under increased strain [5]. Value-based healthcare is currently described as a possible solution for this problem, which aims to improve patient-relevant outcomes relative to the costs [6]. Measuring and improving outcomes in care delivery is a central element of value-based healthcare [7]. The Care Delivery Value Chain is an example of a support tool to describe those activities (e.g. preoperative counselling, shared decision-making) that add value for the patient [8]. One of the current drawbacks of value-based healthcare is that it does not provide a systematic approach to bridge the gap between insights in outcomes and actual improvements in healthcare based on those insights [9].

**Fig. 1:**
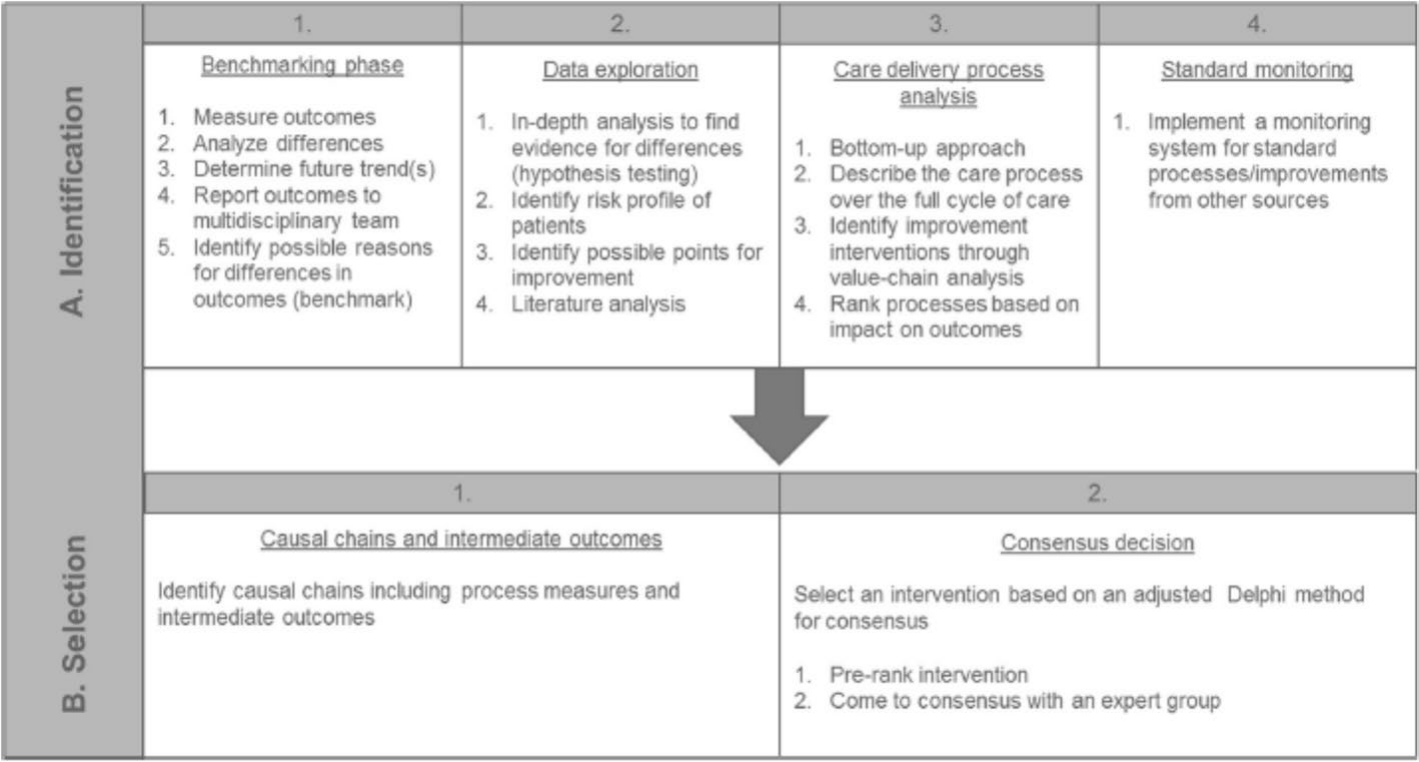
The Intervention Selection Toolbox [10]

The Intervention Selection Toolbox was developed in a case study for cardiac surgery. The purpose was to provide a user-friendly tool for healthcare professionals to identify and select improvement interventions based on insights in outcomes and care delivery processes in and to fill the existing gap between measuring and improving patient-relevant health outcomes [10]. The toolbox was developed for healthcare professionals in general and specifically for multidisciplinary teams. The Intervention Selection Toolbox consists of four steps to systematically identify improvement interventions and two steps to select the improvement interventions with the highest patient value (Fig. 1) [10]. Now that a systematic hands-on approach exists, the obvious next step seems to be to implement this model in the daily practice of health professionals since the Intervention Selection Toolbox has not been widely adopted outside the developmental case study setting.

## Objectives

In this study we aimed to evaluate the implementation and use of the Intervention Selection Toolbox [10] in the daily practice of a hospital setting. Primary endpoint was the number of improvement interventions identified by the Intervention Selection Toolbox. Secondary endpoints are degree of implementation and qualitative evaluation by semi-structured interviews.

## Methods

An evaluation, according to a methodological triangulation study design, was performed in a single large teaching hospital. For a transparent and comprehensive description of the Intervention Selection Toolbox the Template for Intervention Description and Replication (TIDieR) checklist was used to provide the healthcare providers an adequate description of the Intervention Selection Toolbox [11].

### Intervention Selection Toolbox

The Intervention Selection Toolbox was developed to select improvement interventions with a high and positive impact on patient value. For an overview of the Intervention Selection Toolbox, see Fig. 1 [10]. The steps of the toolbox were used in meetings of the quality improvement team following the method Zipfel et al. The toolbox consists of six steps to support consensus decision-making on identification and selection of improvement intervention(s). The first step was to conduct a systematic analysis to identify meaningful differences compared to practice in other hospitals (Benchmarking). The second step consists of data analyses to validate the differences found in the Benchmarking (Data exploration). The third step is to conduct a Care Delivery Process Analysis to identify improvement interventions bottom-up (Care Delivery Process Analysis). The fourth step is to monitor and integrate ongoing overall improvements (Standard monitoring). The aim of this step is to identify other interventions that may influence outcomes of specific patient populations and outcomes. Step five consists of the assessment of the potential improvement impact of the intervention and identification of the causal chains (Causal chains and intermediate outcomes). Finally, the sixth step is the selection of the improvement interventions, which is performed through an adjusted Delphi Method (Consensus decision).

### Study setting

The Intervention Selection Toolbox was used by the colorectal cancer multidisciplinary quality improvement team for decision making in colorectal cancer care in a large non-academic teaching hospital, which is part of the Santeon consortium. Santeon is a Dutch consortium of seven teaching hospitals. With a staff of 35.600 employees, Santeon delivers 11% of the nation’s hospital care volume. Starting in 2010, the seven hospitals have been working together to measure and compare clinical outcomes, costs and process indicators across several patient disease groups including colorectal cancer, as part of the “together better” program [12]. Santeon uses the network’s combined expertise of central data analysts and core team meetings to discuss the observed variation between hospitals. Cross-hospital meetings are followed by hospital-level meetings of multidisciplinary quality improvement teams to discuss possible drivers of observed variation in outcomes relative to other Santeon hospitals. This all results in the Santeon improvement cycle to aim for the best patient care at acceptable costs [12]. The Intervention Selection Toolbox will be implemented as a component of this improvement cycle in the stage of selecting improvement initiatives.

The studied hospital has a total of 750 beds in the hospital, approximately 25 beds for colorectal cancer surgery, a total of 215 annual colorectal cancer procedures in 2019 (*n* = 244 in 2018), and five FTE of colorectal surgeons. In this hospital, value-based healthcare has been practiced since 2015. Colorectal cancer has been organized in a care chain setting since 2019 (Spruijt T, Nat P van der, Bruijne M de: Effects of Care Chains as a matrix-organisation on value-based quality improvements and culture at a Dutch teaching hospital: a qualitative study, Submitted) to achieve logistic optimization. Measurement and improvement of outcomes is a subgoal of the care chain and is represented by a quality improvement team as described by Daniels et al. (Daniels K, Rouppe van de rVoort M, Biesma DH, van der Nat PB: Five years’ experience with value-based quality improvement teams: the key factors to a successful implementation in hospital care, Submitted). The quality improvement team of colorectal surgery consist of members representing the relevant disciplines (surgery, gastroenterology, radiology, anaesthesiology, physiotherapy, dietician, nurses, and pathology). The quality improvement teams get support from a value-based healthcare advisor, a Lean advisor, and a data analyst. There is a daily board of the care chain, which consists of three members; colorectal surgeon, nurse practitioner, and nursing department head, 0.1 FTE each. The daily board is the lead of the quality improvement team. The daily board is formally responsible for quality of care in the care chain. They have weekly meetings to set agendas, goals and maintain communication within the care chain. Colorectal cancer care was selected for this study, because it offered the needed infrastructure, in the form of lateral relationships as described by Galbraith, to integrate the Intervention Selection Toolbox [13]. This study took place during several multidisciplinary meetings in which the Intervention Selection Toolbox was used to guide group discussion and reach consensus on improvement interventions. The group received a handout on the Intervention Selection Toolbox according to the TIDieR checklist and the researcher of this study took participated in the multidisciplinary meetings to guide the members through the use of the Intervention Selection Toolbox if question arose. A non-medical scientific research declaration was obtained from the Medical Research Ethics Committees United (MED-U) of the St. Antonius hospital with reference number: W22.196. The study was in Accordance with non-medical scientific research declaration. All participants gave written informed consent to participate in the study.

### Data collection

Both quantitative data on implementation, through number of improvement initiatives identified by the Intervention Selection Toolbox, and qualitative data through semi-structured interviews were collected. A short overview of methods and used models is displayed in the Supplementary in Table 1.

#### Data collection on implementation of the Intervention Selection Toolbox

For data collection on degree of implementation of the Intervention Selection Toolbox, we assessed the fidelity of the tool. Fidelity summarizes whether the intervention was delivered as intended [6, 7]. From observations of multidisciplinary team meetings implementation every step of the Intervention Selection Toolbox was scored by the researcher on a 4-likert scale with a range of 1–4 (1 = no evidence of implementation and 4 = imbedded in daily practice). The dose (exposure/satisfaction) was studied through qualitative thematic analysis. The reach (participant attendance) of the intervention was not studied, since the toolbox was used in a multidisciplinary quality improvement team where all participants had to engage in the intervention.

#### Data collection on evaluation of the Intervention Selection Toolbox

After implementation, in-depth interviews were conducted with physicians, nurses and other members of the quality improvement team to evaluate the implementation and use of the Intervention Selection Toolbox in daily practice. The interviews were based on a semi-structured interview guide focusing on the key elements of process evaluation (Supplementary Table 2). An interview topic guide was developed based on studies on implementation of quality improvement which identified implementation influencing factors (both facilitators and barriers) [14, 15]. In addition to these studies, the Model for Understanding Success in Quality model was used to take into account relevant contextual factors [16]. A representative sample of professionals was obtained for the semi-structured interviews on the evaluation of the Intervention Selection Toolbox, which included the medical leader, gastrointestinal surgeons, gastroenterologists, nurse practitioners, the nursing leader, paramedical staff (e.g. radiologist). A thematic framework approach was used in the analysis of the interviews. Direct observations were conducted during all the meetings of the quality improvement team. Interviews were audio recorded and transcribed verbatim. Interviews were undertaken until data saturation occurred [17].

### Statistical analysis

Descriptive statistics were used to report data. Sequential analysis was used for data collection and analysis. As for the qualitative analysis, the transcribed interviews were analysed through a thematic analysis which included coding and categorizing patterns and themes in the data. Two researchers (J.V. and M.F.) independently coded the transcribed interviews into a frame. Several meetings took place between JV and MF to reach consensus on coding of the transcribed interviews. A third independent reviewer (B.S.) analysed codes and transcribed interviews to identify key themes through thematic framework approach. Peer debriefing was performed after realization of the different themes with the researchers (J.V. and M.F.). The software ATLAS.ti (Version 8.4.5) was used for the qualitative data analysis. Final themes were not identified in advance.

## Results

Results of this study are divided between (1) implementation and (2) evaluation of the Intervention Selection Toolbox in daily practice of a multidisciplinary quality improvement team for colorectal cancer care in a large teaching hospital.

### Implementation of Intervention Selection Toolbox

As for implementation of the Intervention Selection Toolbox, benchmarking was fully implemented in daily practice. Data exploration, care delivery process analysis and consensus decision were all systematically implemented (according to the protocol of the toolbox). However, standard monitoring was discussed infrequently and was not considered as a standard agenda item. Also, defining causal chains and intermediate outcomes were not performed during the observed meetings. The degree of implementation for each step of the toolbox (fidelity) is summarized in Table 1.

**Table 1:** Degree of implementation (fidelity) of the intervention selection toolbox in daily practice

**Intervention selection toolbox component**	**Degree of implementation (fidelity)**	**Comments**
**Step A.1:** **Benchmarking**	4- Imbedded in daily practice	Imbedded in daily practice, cyclical benchmarking between seven Santeon hospitals on colorectal cancer care outcomes.
**Step A.2:** **Data exploration**	3- Systematical	Part of the cyclical Santeon quality improvement program for colorectal cancer. Discussed in local meetings of the 7 Santeon hospital quality improvement teams and during the six-monthly Santeon meeting in which the local teams come together.
**Step A.3:** **Care delivery process analysis**	3- Systematical	Systematic care delivery process analysis through several one hour meetings with relevant disciplines.
**Step A.4:** **Standard monitoring**	2- Some evidence of implementation	Infrequently discussed during weekly meetings of the colorectal cancer pathway leadership. Monitoring was not a standard agenda item. However, it was monitored in writing.
**Step B.1:** **Causal chains and intermediate outcomes**	1-No evidence of implementation	Not implemented.
**Step B.2:** **Consensus decision**	3- Systematical	Consensus decision making took place in weekly meetings of the colorectal cancer care pathway leadership which was comprised of a colorectal surgeon, specialized nurse and manager.

- A: Identification of improvement potential
-
  1. Benchmarking outcomes
    Our hospital participated in the benchmarking between the Santeon hospitals, which was already performed yearly as part of the Santeon improvement cycle [18]. In our hospital the benchmark data was collected by a data analyst of the quality improvement team. The data was discussed every two months by the quality improvement team. Also, delegations of each Santeon hospital (usually a surgeon and a data analyst) met every six months in a Santeon wide meeting to discuss the data. Based on these benchmark results, there was no motive for improvement initiatives for our hospital. However, there were some discrepancies relating to up- and downstaging of tumour classification after radiologic imaging (according to the TNM classification), which the team decided to further explore in the next step of the toolbox.
  2. Data exploration
    Data exploration is performed to investigate discrepancies or differences that were found in benchmarking step. This step is performed regularly after the benchmarking step. This means that data exploration is performed every two months internally and every six months in a Santeon wide meeting. After the benchmarking of this study the discrepancies in tumour classification and radiologic imaging were investigated. In our hospital the medical files, including radiologic imaging and pathology reports, of the patients who underwent tumour staging, were re-examined by the radiologist and the pathologist. The data exploration was done by the data analyst and discussed in several quality improvement team meetings.
  3. Care delivery process analysis
    The care delivery process encompasses the full cycle of care for colorectal cancer within our hospital. The care delivery process was subdivided into diagnostics, prehabilitation prior to surgery, outpatient clinic and surgical ward and palliative treatment [6]. During four process analysis meetings, the members of the quality improvement team aimed to identify possible quality improvement interventions by a bottom-up approach analysis at each step in the care delivery process. The full cycle of care in the hospital was subdivided in four parts. Each process analysis meeting consisted of members of the quality improvement team and medical personnel related to that subdivision (e.g. physicians, nurses, dietitian etc.). Possible quality improvement interventions were rated on two dimensions: impact on outcomes and effort required to implement. A total of 80 quality improvement interventions were identified during the four process analysis meetings. These were divided in: high impact/low effort (*n* = 32), high impact/high effort (*n* = 47) and low impact/low effort (*n* = 2).
  4. Standard monitoring
    As opposed to the fourth step of the initial Intervention Selection Toolbox, monitoring and reporting on ongoing improvements was infrequently scheduled during weekly meetings of the daily board of the care chain colorectal cancer, due to limited time. Monitoring of ongoing improvement was not scheduled as a standard agenda item due to the number of agenda points. When discussed, the lead of the multidisciplinary team was in charge of updating on the progress of the improvement interventions. The data analyst complemented updates with data on the improvement and final outcomes. Although not standardly reported, ongoing improvement initiatives were monitored in writing in the background by the data analyst.

- B: Selection of an improvement interventions
-
  1. Causal chains and intermediate outcomes
    This step of the Intervention Selection Toolbox, was not performed in our hospital. During the multidisciplinary team meetings was discussed whether the interventions could have causal relations to other parts of the care delivery process and if there were other possible factors to influence the final outcomes. However, the proposed interventions had a clear causal relation to final outcomes, so there was no need for causal chain description.
  2. Consensus decision
    In our hospital, improvement interventions were divided in smaller and larger scale improvement interventions before reaching a consensus decision. The smaller scale improvement interventions were decided on during the weekly meetings of the daily board, outside the consensus decision meetings with the full quality improvement team. Larger scale interventions with high improvement potential and a greater number of stakeholders were decided on during consensus decision meetings, where all stakeholders of the quality improvement team were present. In case of disagreement further discussion took place until consensus was reached. The consensus decision led to two high potential improvement interventions, which were selected from the 80 initial quality improvement interventions.

### Evaluation of the Intervention Selection Toolbox

Thematic saturation was reached after conducting ten semi-structured interviews. These were ten members, all members of the quality improvement team, from a wide range of medical specialists, nurses and paramedical staff. Age ranged from 28 to 61 years, clinical experience ranged from 6 to 38 years. Highest educational attainment ranged from vocational to academic doctorate.

Analysis of the interviews produced three major themes which were associated with implementation of the Intervention Selection Toolbox, and were consistently expressed by participants. Together these themes form a thematic framework, which illustrates the view of the members of the quality improvement team on the use of the Intervention Selection Toolbox in daily practice.

### Theme 1: impact and outcomes

All participants considered the Intervention Selection Toolbox as added value to evaluate and improve the care process in the healthcare system in colorectal cancer. Participants considered the Intervention Selection Toolbox as a suitable way to gain additional insights on each aspect of a multidisciplinary approach and on each contributing team. One participant mentioned, *“…process analysis shows that people who are involved do not know what happens in other parts of the process most of the time.”* Majority of participants also agreed that use of the Intervention Selection Toolbox could create more support and involvement. For example, one person shared, *“It also creates a form of commitment to engage in the whole process.”* However, a few of the participants expressed doubts on the abstract character of the Intervention Selection Toolbox and addressed the need for concrete goals and measurements. As a result of a lack of concrete goals, the impact on daily practice was questioned by some participants, *“I am not sure if this has influence on my daily practice.”*

### Theme 2: communication and decision making

All participants described clear communication, repetition of the benchmarking and standard monitoring as key items to assure success in implementation of the Intervention Selection Toolbox. In terms of repetition of the process analysis, participants agreed on the added value of benchmarking, standard monitoring and bottom-up process evaluation. For instance, one participant stated, *“…repeat once a year and look back to see what was accomplished in this period. What is the current state of affairs? And what are goals of the coming year or years?”* Other participants also stated the need of evaluation of daily practice in the form of standard monitoring and a clear feedback loop on improvement measurements to the rest of the team to contribute to commitment and further improvement of the healthcare, *“We need good evaluation* [benchmarking and standard monitoring] *and, more importantly, adequate feedback of the evaluation to the rest of the team to preserve motivation.”*

### Theme 3: resource availability and time management

The majority of participants described high motivation for innovation in their department. For instance, one participant stated, *“I think that the attitude is really great … employees are constantly searching for improvement.”* Another participant said, *“There is a mutual culture of support on the subject of innovation in the department.”*

However, participants also described the hospital setting as a time and energy consuming organization to make changes. For instance, *“… if we want to make big changes, it costs a lot of energy of all people involved.”* Reasons for this were constrains due to time limits and increasing task load amongst employees. One of the participants stated, *“…so many departments and stakeholders call for different improvements in the hospital. There is weariness because of all the needed registration.”* Another participant argued time limits as a reason for less progression: *“On the other side it is a bit much … some projects have little progression because it is just too much at once.”* Time overall was stated to be a key element by the majority of participants to achieve feasibility of the Intervention Selection Toolbox,* “…they needed to have time to engage in the process. That is the most important condition.”*

## Discussion

The results of this study show that most steps of the Intervention Selection Toolbox have been implemented systematically in a quality improvement team of colorectal cancer. The involved hospital personnel viewed the Intervention Selection Toolbox as a valuable tool for the identification and selection of quality improvement efforts.

As for the extent of implementation (fidelity), most steps were successfully and systematically implemented (Table 1). Standard monitoring and causal chain analysis were steps of the model that were not systematically implemented, opposed to the recommendations that the original development study of Zipfel et al. made [10]. Standard monitoring was often left out during the meeting due to time restrictions. However, since the qualitative interviews showed the value of communication and feedback in the use of the Intervention Selection Toolbox, standard monitoring should be performed during meetings if the available time allows it. If not, it should be monitored in the background. In our organisation the causal chain step was often considered irrelevant and therefore skipped, since causal relations were clear. This suggests that specific components of the Intervention Selection Toolbox may differ in relevance between organisations and medical specialties. Causal chain analysis may be considered as an optional step in this toolbox, as shown in the adjusted version of the Intervention Selection Toolbox in the Supplementary Figure 1. Each organization should evaluate relevance and feasibility of these components for their own organization. The participation of the personnel in the use of the Intervention Selection Toolbox was sufficient and regular thanks to the already existing quality improvement infrastructure, such as weekly, monthly and bi-annual meetings. Also, the exposure of the entire team to the Intervention Selection Toolbox was provided by the four process analysis meetings which were attended by a large representation of the different involved medical professionals.

Qualitative analysis from the interviews showed implementation to be more complex than expected and universally applicable. For example, needed organizational structure is a condition of the toolbox which made implementation in daily practice more complex. The quality improvement team of colorectal cancer has an organizational structure which matches the lateral relations strategy described by Galbraith [13]. The lateral relation strategy cuts across lines of authority and moves the decision making down in the organization. The use of this strategy in the organizational structure may benefit the Intervention Selection Toolbox, since the medical professionals themselves can come up with proposed interventions and select the interventions with the highest impact on patient outcomes. Previous research on implementation of value based healthcare also suggests that formal responsibility of care and improvement increases the involvement of team members (Daniels K, Rouppe van de rVoort M, Biesma DH, van der Nat PB: Five years’ experience with value-based quality improvement teams: the key factors to a successful implementation in hospital care, Submitted).

The team experienced the Intervention Selection Toolbox as a relatively intensive process which results in limited number of interventions, since only high-impact improvement interventions are selected in the model [10]. Therefore, we identified time and personnel availability as restricting factors in the implementation of the Intervention Selection Toolbox. This is in line with current literature regarding heavy workloads and time related pressure amongst hospital personnel compared to the general population [19, 20]. These findings contribute to the previously described readiness of an organization as a key item to success and improvement in outcomes [21]. Also, during the interviews researchers noticed that participants linked evaluation and success of the Intervention Selection Toolbox directly to the success and evaluation of the selected interventions. As such, the Intervention Selection Toolbox which is a component of the improvement cycle, could not be judged separately from the improvement cycle. The improvement cycle also includes the implementation of the interventions, which is beyond the scope of the Intervention Selection Toolbox.

This study provides new insights on the use and important themes that should be considered before and during implementation of the Intervention Selection Toolbox compared to the previous study of Zipfel et al. [10]. Firstly, the cardiac department that used the Intervention Selection Toolbox in the study of Zipfel et al., involved less stakeholders in their multidisciplinary team and the range of functions were less broad compared to this study. Besides cardiac valve treatment and colorectal cancer are two different diseases with their own pathology, which may influence the added value for patients of the Intervention Selection Toolbox. Also, Zipfel et al. chose to develop the Intervention Selection Toolbox in a stakeholder session only, whereas this study evaluated the tool in the daily practice of a large multidisciplinary team that is part of a care chain [10]. Type of disease (e.g. chronic or acute) and amount of stakeholders that are involved in the multidisciplinary setting could affect the efficiency of the Intervention Selection Toolbox. This evaluation provides information about Intervention Selection Toolbox that could increase the added value for patients, since its’ effect, potential facilitators and barriers are identified and may be addressed to optimize its’ use.

This study is strengthened by the methodological triangulation, since it allows to evaluate the Intervention Selection Toolbox from a quantitative and qualitative view which are both important in evaluation. However, this study also poses a number of possible limitations. Firstly, not all members of the colorectal cancer multidisciplinary quality improvement team were acquainted with involved terminology and theory regarding value-based healthcare and the Intervention Selection Toolbox. However, during implementation and evaluation the different steps of the toolbox were adequately performed and evaluated by all members, even though exact terminology was not used. This may suggest that the Intervention Selection Toolbox can be performed, even though not all involved personnel is fully aware of the underlying theoretical knowledge on value-based healthcare. Another explanation would be that theoretical knowledge on value-based healthcare can positively influence efficiency of the Toolbox. Secondly, in this study we aimed to evaluate the use of the Intervention Selection Toolbox, but the use of the tool may not be judged separately from the success of the interventions. Also, the Intervention Selection Toolbox was initially developed to become a standard element of an ongoing quality improvement cycle. However, in the context of this study, the Intervention Selection Toolbox was only evaluated in an one six month improvement cycle. Even though most elements of the Intervention Selection Toolbox were considered useful by the quality improvement team members, it is unclear to what extent the Intervention Selection Toolbox has been truly adopted as part of the continuous ongoing improvement cycle.

It would be interesting for future research to assess what organizational structure allows the best efficiency and time management in the improvement cycle and to evaluate the use of the Intervention Selection Toolbox in a chronic disease setting with a broad multidisciplinary team. In this future research, themes that are identified in this study (such as time constraints and clear communication loops) should be addressed. Thereafter, the next step would be to perform an implementation study.

## Conclusion

Our study shows that the Intervention Selection Toolbox has been used with moderate success in a quality improvement team for colorectal cancer. The toolbox is viewed by personnel as a useful tool to assess health outcomes and systematically select improvement initiatives, but appropriate organizational structure and time management are key items to assure maximal success and efficiency. Topics of interest for future research are identification of the best organizational structure and optimal time management for the Intervention selection toolbox in a chronic disease setting with a broad multidisciplinary team.

## Supplementary Information

**Additional file 1: Supplementary Table 1.** Methods overview.

**Additional file 2: Supplementary Table 2.** Interview guide semi-structured interview.

**Additional file 3: Supplementary Figure 1.** Adjusted Intervention Selection Toolbox*.*

**Additional file 4: Supplementary file 4.** Raw data regarding interviews.

## Data Availability

All data generated or analyzed during this study are included in this published article supplementary file 4.

## References

[1] Berwick DM, Nolan TW (1998). Physicians as leaders in improving health care: a new series in Annals of Internal Medicine. Ann Intern Med.

[2] Berwick DM (2004). Lessons from developing nations on improving health care. BMJ.

[3] Rubenstein LV, Mittman BS, Yano EM, Mulrow CD (2000). From understanding health care provider behavior to improving health care: the QUERI framework for quality improvement. Quality Enhancement Research Initiative. Med Care.

[4] Dixon-Woods M, McNicol S, Martin G (2012). Ten challenges in improving quality in healthcare: lessons from the Health Foundation’s programme evaluations and relevant literature. BMJ Qual Saf.

[5] Bodenheimer T (2005). High and rising health care costs. Part 1: seeking an explanation. Ann Intern Med.

[6] Porter ME (2010). What is value in health care?. N Engl J Med.

[7] Porter ME (2008). Value-based health care delivery. Ann Surg.

[8] Kim JY, Farmer P, Porter ME (2013). Redefining global health-care delivery. Lancet (London, England).

[9] van der Nat PB. The new strategic agenda for value transformation. Health Serv Manage Res. 2022;35(3):189–93. 10.1177/09514848211011739. 10.1177/09514848211011739 PMC9277321 33900128

[10] Zipfel N, Groenewoud AS, Rensing BJWM, Daeter EJ, Dijksman LM, Dambrink JHE (2020). Selecting interventions to improve patient-relevant outcomes in health care for aortic valve disease - The Intervention Selection Toolbox. BMC Health Serv Res.

[11] Hoffmann TC, Glasziou PP, Boutron I, Milne R, Perera R, Moher D (2014). Better reporting of interventions: Template for intervention description and replication (TIDieR) checklist and guide. BMJ.

[12] Santeon, BCG. Hospitals Make Health Care Work. 2018; Available from: https://www.bcg.com/publications/2018/how-dutch-hospitals-make-value-based-health-care-work.aspx

[13] Galbraith JR (1974). Organization Design: An Information Processing View. Interfaces (Providence).

[14] Evans-Lacko S, Jarrett M, McCrone P, Thornicroft G (2010). Facilitators and barriers to implementing clinical care pathways. BMC Health Serv Res..

[15] Bradley EH, Nembhard IM, Yuan CT, Stern AF, Curtis JP, Nallamothu BK (2010). What is the experience of national quality campaigns? Views from the field. Health Serv Res.

[16] Kaplan HC, Provost LP, Froehle CM, Margolis PA (2012). The Model for Understanding Success in Quality (MUSIQ): building a theory of context in healthcare quality improvement. BMJ Qual Saf.

[17] Guest G, Namey E, Chen M (2020). A simple method to assess and report thematic saturation in qualitative research. PLoS ONE.

[18] El Hadj Amor EA, Ghannouchi SA (2017). Towards KPI-Based Health Care Process Improvement. Procedia Comput Sci.

[19] Alpert JS (2008). Physician depression. Am J Med.

[20] Tsai Y-C, Liu C-H (2012). Factors and symptoms associated with work stress and health-promoting lifestyles among hospital staff: a pilot study in Taiwan. BMC Health Serv Res.

[21] Kampstra NA, Zipfel N, van der Nat PB, Westert GP, van der Wees PJ, Groenewoud AS (2018). Health outcomes measurement and organizational readiness support quality improvement: a systematic review. BMC Health Serv Res.

