# Supplementary material for: The Intervention Selection Toolbox to improve patient-relevant outcomes: an implementation and qualitative evaluation study in colorectal cancer surgery
Source: BMC Health Serv Res. 2023 Apr 6;23:345. doi: 10.1186/s12913-023-09264-3 (PMC10080915; doi:10.1186/s12913-023-09264-3)
Supplement: Supplementary file 1 — Additional file 1: Supplementary Table 1. Methods overview. [file 12913_2023_9264_MOESM1_ESM.docx]

| *Data collection* | *Assessment strategy* | *Used models* |
| --- | --- | --- |
| *1. Data collection on implementation of the Intervention Selection Toolbox* | *Description of implementation extent* | *Template for Intervention Description and Replication*  *(TIDieR checklist)* |
| *2. Data collection on evaluation of the Intervention Selection Toolbox* | *In depth Semi-structured interviews* | *Model for Understanding Success in Quality model* |

Supplementary Table 1. Methods overview
