# Supplementary material for: The Intervention Selection Toolbox to improve patient-relevant outcomes: an implementation and qualitative evaluation study in colorectal cancer surgery
Source: BMC Health Serv Res. 2023 Apr 6;23:345. doi: 10.1186/s12913-023-09264-3 (PMC10080915; doi:10.1186/s12913-023-09264-3)
Supplement: Supplementary file 2 — Additional file 2: Supplementary Table 2. Interview guide semi-structured interview. [file 12913_2023_9264_MOESM2_ESM.docx]

| **Subject** | **Questions** | **MUSIQ model category** |
| --- | --- | --- |
| Which role do you play in the quality improvement team? |  | inapplicable |
| Process analysis | What is your opinion on process analysis using the Intervention Selection Toolbox?   - Does this process analysis add value compared to the current approach? - Is implementation of this process analysis in current approach achievable? - Would you like to adjust components of this process analysis? | inapplicable |
| Causal chain analyses | What is your opinion on causal chain analysis using the Intervention Selection Toolbox?   - Does this causal chain analysis add value compared to the current approach? - Is implementation of this causal chain analysis in current approach achievable? - Would you like to adjust components of this causal chain analysis? | inapplicable |
| Decision making | What is your opinion on the consensus decision approach using the Intervention Selection Toolbox?   - Does this consensus decision approach add value compared to the current approach? - Is implementation of this consensus decision approach in current approach achievable? - Would you like to adjust components of this consensus decision approach? | inapplicable |
| Implementation intervention | Which actions were undertaken to implement the Intervention Selection Toolbox?   - How were the actions defined? - Who were involved? - How were all professionals, who are involved in the process, informed during implementation? | inapplicable |
| Support from local hospital | How did the hospital support the implementation project?   - What was the roll of the supporting personnel (e.g. quality assurance officers)? - What resources were available? (e.g. time, personnel, training) | QI support and capacity: resource availability |
| Senior management | Was the senior management (e.g. local hospital board) involved in some manner? | Organization: QI leadership |
| Appreciation for improvement | What is the attitude, in terms of appreciation, in your hospital and team on quality improvement (e.g. Valued-based Health Care approach)? | Microsystem: culture supportive of QI |
| Willingness to change | - Is there willingness to change in the quality improvement team? - Is change stimulated and facilitated by the medical leader? | Microsystem: motivation to change |
| Team members | Who were involved in the quality improvement team?   - Who was the team leader? - Did you know your co-workers already? - Did anyone have previous experience on quality improvement projects? - How would you describe the collaboration? | QI team: team diversity, team tenure, prior QI experience, |
| Skills | Did the quality improvement team have the right skills to implement the quality improvement intervention? | QI team: QI skills |
| Compensation | - Do team members receive compensation in any way for their efforts? - Are there financial incentives? | QI support and capacity: resource availability |
| Data infrastructure | Is there a system available to extract the needed data? | QI support and capacity: data infrastructure |
| Impact | What was the impact of the implementation of the intervention on the clinical practice? | inapplicable |
| Unexpected effects | - Were there unexpected effects? - Were there unexpected factors which facilitated or opposed the intervention? - Did workload increase by the use of the intervention? | inapplicable |
| Outro | Are there any other questions that should have been asked to understand the implementation process more? | inapplicable |

Supplementary Table 2. Subjects in semi-structured interview
