# Supplementary figures and images for: The Intervention Selection Toolbox to improve patient-relevant outcomes: an implementation and qualitative evaluation study in colorectal cancer surgery
Source: BMC Health Serv Res. 2023 Apr 6;23:345. doi: 10.1186/s12913-023-09264-3 (PMC10080915; doi:10.1186/s12913-023-09264-3)

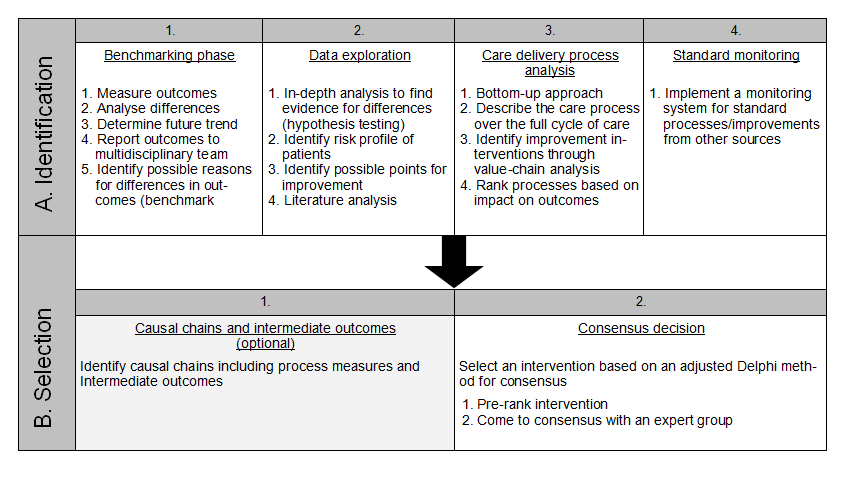


*Supplementary Figure 1. Adjusted Intervention Selection Toolbox*

Supplement: Supplementary file 3 — Additional file 3: Supplementary Figure 1. Adjusted Intervention Selection Toolbox. [file 12913_2023_9264_MOESM3_ESM.docx]
