# Supplementary material for: The Intervention Selection Toolbox to improve patient-relevant outcomes: an implementation and qualitative evaluation study in colorectal cancer surgery
Source: BMC Health Serv Res. 2023 Apr 6;23:345. doi: 10.1186/s12913-023-09264-3 (PMC10080915; doi:10.1186/s12913-023-09264-3)
Supplement: Supplementary file 4 — Additional file 4: Supplementary file 4. Raw data regarding interviews. [file 12913_2023_9264_MOESM4_ESM.docx]

**The IST model**

**Flowchart IST**

**Implementation**

Step 1:

Benchmarking

Step 2:

Data exploration

Step 3:

Care delivery process analysis

Step 4:

Standard monitoring

**A: Identification of improvement potential**

Step 1:

Causal chains and intermediate outcomes

Step 2:

Consensus decision

**B: Selection of an improvement intervention**

Identify differences in outcomes among hospitals

Validate or confirm hypotheses of Step 1

Bottom-up identify intervention(s)

Identify ongoing interventions with impact on same outcomes

Estimate potential impact of intervention(s)

Make final selection of intervention(s)

**Steps**

**Goals**

2 VBHC cycles, every year, using existing care pathways as framework

VBHC cycle, every 6 months, meeting 2

VBHC cycle, every 6 months, meeting 1

Every meeting within VBHC cycle, every 2 months

VBHC cycle, every 6 months, meeting 3

VBHC cycle, every 6 months, meeting 3

VBHC IST

Meeting 3

Meeting 2

Meeting 1

Step 1:

Causal chains and intermediate outcomes

Step 2:

Consensus decision

Step 4:

Standard monitoring

Step 3:

Care delivery process analysis (1x per 2 VBHC cycli)

Step 2:

Data exploration

Step 1:

Benchmarking

**Part A: Identification of improvement potential**

Step 1: Benchmarking

Goal: Identify differences in outcomes among hospitals and formulate hypotheses for possible causes of interesting differences that have been observed.

Implementation: Is currently embedded in the VBHC improvement cycle. Every 6 months during meeting 1 results of benchmarking of the 7 Santeon hospitals are shown. Data is collected by the data analysts of each hospital based on the outcome set which is developed by the lead Santeon hospital and edited and eventually approved by the other 6 Santeon hospitals.

Step 2: data exploration

Goal: Validate or confirm hypotheses of Step 1

Implementation: Is currently embedded in the VBHC improvement cycle. Every 6 months during meeting 2 the results of the data exploration of our own hospital is shown. Data exploration is performed by the data analist of our own hospital. Hypotheses formulated in meeting 1 are confirmed or rejected.

Step 3: Care delivery process analysis (CDPA)

Goal: Bottom-up identify intervention(s)

Implementation: CDPA is currently not performed in the VBHC improvement cycle. This step will be added to the VBHC cycle and will be performed every year (i.e. every 2 6-month cycli) in meeting 2. This moment in the VBHC cycle has been chosen because data from step 1 and 2 (presented in meeting 1 and 2 respectively) will also be considered during the CDPA. Meeting 2 is currently assigned for data exploration and identifying and selecting improvement interventions. Data exploration will remain a part of meeting 2, however identifying and selecting improvement interventions will be postponed to meeting 3. This provides us with time to perform the CDPA, it is imperative that representatives of all involved specialties and paramedics are involved in the CDPA.

Existing care pathways will be used whenever possible as a framework. The multidisciplinary team will use a scoring tool based on the CDVC framework to score each process component per treatment based on the following criteria: (1) impact on patient-relevant outcomes, (2) room for improvement (taking into account data from step 1 and 2), and (3) feasibility to improve.

Potential improvement interventions will be formulated by the team based on the scoring tool and will be linked to one of the outcomes formulated in step 1. After a compilation and evaluation of the improvement interventions, we will present a list of interventions in meeting 3 to the entire multidisciplinary team.


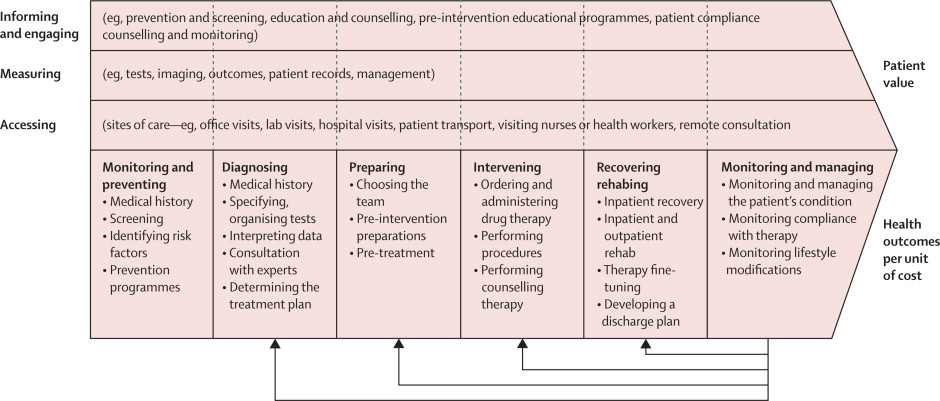


Step 4: Standard monitoring

Goal: Identify ongoing interventions with impact on same outcomes

Implementation: This step is currently not routinely performed in the VBHC improvement cycle but will be added as a standard component of every 2-month meeting. Members of the multidisciplinary team will make an inventory of ongoing interventions in their respective specialties or wards and will report these interventions during every meeting. This monitoring step is needed to identify potential ongoing improvement interventions with impact on the same outcome measures as identified in Step 1 and 2. What is also needed is an overview of ongoing improvement interventions to be able to judge the added value of the improvement interventions resulting from Step 1-3. We regularly updated the standard monitoring whenever new improvement interventions were started up at the primary hospital. A list of ongoing improvement interventions will be maintained.

**Part B: Selection of an improvement intervention**

Step 1: Causal chains and intermediate outcomes

Goal: Estimate potential impact of intervention(s)

Implementation: This step is not performed in the VBHC improvement cycle and will be added to meeting 3. To estimate the potential impact of the improvement interventions on the outcome measures, a causal chain analysis will be performed. This is the path from improvement intervention to outcome measure. In between the intervention and a patient-relevant outcome are intermediate outcomes, which are outcomes that are impacted more directly by the intervention. Intermediate outcomes were relevant for monitoring the impact of an improvement intervention. They also allow for proving an effect when the impact on the outcome measures would be too small to measure statistically significant impact. The results of part A formed the basis for this step.

Two members of the multidisciplinary team and a medical specialist will rank the results according to relevance. Relevance will be scored on a three-star scale from limited to high impact with the following criteria which will be added to an overall score: (a) impact on the outcome measure, (b) technical and practical feasibility and (c) feasibility in terms of costs. The aim of this ranking is to narrow down a pre-selection to offer a sharper scope of the possible improvement interventions.

Step 2: Consensus decision

Goal: Make final selection of intervention(s)

Implementation: The multidisciplinary team will be asked to score the improvement interventions once with the information on the causal chains according to the impact on patient-relevant outcomes during a team meeting. The multidisciplinary team will be given the chance to revise their choice at the end of the first round of prioritization. The final decision is made at the end of the meeting and follow-up meetings are organized to further design implementation of the intervention.

Inserting questionnaires from the current VBHC method:

Show 3 meetings, agenda per meeting in which the 6 steps have already been incorporated.

PowerPoint presentation in which the new working method is clearly stated (agenda per meeting, example of a process analysis, etc.). With an accompanying questionnaire about the presentation.

Interview set-up

| **Factor** | | | | | **Definition** | **Code** |
| --- | --- | --- | --- | --- | --- | --- |
| **External environment →** | | | | |  |  |
|  | | | | External motivators | Environmental pressures and incentives that stimulate the organisation to improve its performance and quality in the area of focus of this QI project | 1 |
|  | | | | Project sponsorship | Substantial and meaningful contributions of personnel, expertise, money, equipment, facilities, or other important resources from outside entities (external to the organisation) with formal relationships with this QI project | 2 |
| **Organization** | | | | |  |  |
|  | | | QI leadership | | Senior management’s (CEO, COO, CMO, Senior VP, Board of Directors) governance-guidance, support, oversight, and direction setting-of improvement efforts | 3 |
|  | | | Senior leader project sponsor | | Senior leader commitment to champion and support this QI project | 4 |
|  | | | Culture supportive of QI | | Values, beliefs, and norms of an organisation that shape the behaviours of staff in pursuing QI | 5 |
|  | | | Maturity of organisational QI | | Sophistication of the organisation’s QI programme | 6 |
| **QI support and capacity** | | | | |  |  |
|  | | | Data infrastructure | | Extent to which a system exists to collect, manage, and facilitate the use of data needed to support performance improvement | 8 |
|  | | | Resource availability | | Degree to which financial support for QI, including allocation of resources and staff time, is provided | 9 |
|  | | | Workforce focus on QI | | Degree to which the organisation develops the workforce through training and engages them in QI through reward systems and expectation setting | 10 |
| **Microsystem** | | | | |  |  |
|  | | QI leadership | | | Microsystem leadership capacity for improvement and degree to which they are personally involved in supporting and facilitating improvement efforts | 11 |
|  | | Culture supportive of QI | | | Values, beliefs, and norms present in the microsystem that emphasise teamwork, communication, freedom to make decisions, and commitment to improve | 12 |
|  | | Cooperation in QI teams | | |  | 12+ |
|  | | Capability for improvement | | | Microsystem staff’s ability to use QI methods for change | 13 |
|  | | Motivation to change | | | Extent to which microsystem staff members have a desire and willingness to improve performance in this area of focus | 14 |
| **QI team** | | | | |  |  |
|  | | Team diversity | | | Diversity of team members with respect to professional discipline, personality, motivation, and perspective | 15 |
|  | | Physician involvement | | | Contribution of physicians to the QI team efforts | 16 |
|  | | Subject matter expert | | | One or more team members is knowledgeable about the outcome, process, or system being changed | 17 |
|  | | Team tenure | | | Team members have worked together as a team before | 18 |
|  | | Prior QI experience | | | Prior experience with QI | 19 |
|  | | Team leadership | | | Team leader’s ability to accomplish the goals of the improvement project through guiding the actions of the QI team | 20 |
|  | | Team decision-making process | | | Team engages in well-designed decision-making practices | 21 |
|  | | Team norms | | | Team establishes strong norms of behaviour related to how work is to be carried out and how goals are to be achieved | 22 |
|  | | Team QI skill | | | Team’s ability to use improvement methods to make changes | 23 |
| **Miscellaneous** | | | | |  |  |
|  | Trigger | | | | Presence of a specific event (positive or negative) that stimulates a new emphasis on improving quality in the area of focus of a given QI project | 24 |
|  | Task strategic importance to the organisation | | | | Work perceived as part of the organisation’s strategic goals | 25 |
| **Not included in MUSIQ model** | | | | |  |  |
|  | | What part do you play in the QI team | | |  | 26 |
|  | | Process analysis IST model | | |  | 27 |
|  | | Causal chain analysis IST model | | |  | 28 |
|  | | Decision making process IST model | | | Over model Nina IST | 29 |
|  | | Cooperation in QI team | | |  | 12+ |
|  | | Implementation activity | | | Which activities were undertaken to implement an intervention? | 31 |
|  | | Impact on outcomes | | | What was the effect of the intervention on clinical care? | 32 |
|  | | Unexpected effects | | | Were there unexpected side effects of the intervention? Were there unexpected factors which facilitated or hampered the implementation of an intervention? | 33 |
|  | | Other | | | Not defined | 34 |

# Interviews (raw data)

m

The first question. What role do you play in the improvement team and in the colorectal cancer care chain?

a

Well, I'm, so to speak, the medical director. So basically I pick up all the medical stuff.

Of course I also work in the chain. So I can also think of what is useful to improve.

m

Yes.

a

And also if people... The intention is actually for people to come up with initiatives. And that we then encourage them to get started with it. And that role, I do.

m

Okay. Clear. And the process analysis during the lunch meetings, were you there in the end?

a

No. But I have now... there was another meeting like this last Tuesday. And I was there.

m

Oh, so you've been through one. And what did you think of that process analysis?

a

Well, in itself uh is that... Last time people said what kind of things they run into. And then what they could do about it. But which is immediately a disadvantage, there were not that many people present last Tuesday. And that's always the problem.

m

Yes. Yes,

a

The involvement of the people. And yes, that's always difficult, isn't it? You know that too, from what you've done to me.

m

That's right.

a

Yes. And it's for people, it comes with it, huh? That is always difficult. But what did you do with that improvement of the dismissal procedure, eh? a, that's just nice to pick something up. But the problem is always that people have to do that in the workplace. You're very dependent on having a few people who just follow it up and say something about it when someone doesn't apply it. In my experience, that often takes a number of years before something really changes.

M

De mensen moeten het nut ervan inzien en je moet natuurlijk ook draagvlak creëren.

a

But even if they see the point and you create support. Then they still find it easy to continue in the old way, automatic pilot I notice.

m

Yes, habits are hard to change.

a

Yes, and a lot of research has actually been done on that and it has to do with that. And that people can then say in conversation with you: I completely agree that we should do that. And meanwhile they don't. And then you have to point it out to them again. And then you have to say oh that's what we agreed. Yes. I think I'm answering several questions now. But..

m

It doesn't matter, this is all useful data.

uhm that process analysis. Is that something you think could be very helpful? Something we should do more often?

a

Yes, I think that process analysis is fine in itself. Only, you know, I often just do that in my head. I know what is possible anyway. What's relevant. What does that mean? But what's especially good about that is that you look at it with a group. And then you pay attention to it again. Then you might create a little more support. I don't need it as a person. Now, for example, that that CRP has to be faster, I have that as a person, I don't need that. But. I do see that it helps in a group to keep you busy with that. But it can sometimes be a threshold for people like oh, then I have to fill in process analysis like that, you know really. It's such a hassle, takes time. And why so formal?

m

Yes.

a

So yes. It goes both ways, doesn't it. So we have gone through the entire care path for everything that could be improved. But that drives you crazy. That was with someone else. So that was a long time ago.

m

Yes. So that process analysis has pros and cons, if I understand correctly.

a

Yes. Yes. So it has the advantage that a lot of people are there. It is also a kind of involvement. And it actually creates a kind of obligation. Uh I think so. So that's very nice. But it also takes a lot of time. uh. Yeah, fine. I think in the end, I think it's better if we do.

m

Yes, clearly. And then another question about decision making. Uhm within the healthcare chain, but also more generally in the hospital. If an improvement project is introduced, how does it go? Is it easy, is it difficult? is that a transparent process or does it happen in all different places where decisions are made where people are not really aware of each other. How are your experiences with that?

a

Well, I think we do that with those brainstorming sessions, fine. Because everyone is involved in that. Yeah look, if you don't show up, that's up to you. But after that, see if we said we're going to do it. Then we will come back to it at other meetings. But then it's just decided, we're going to do that. Then we will come back to that at the next meeting.

How did that go? Those are all important things to do. It's all a bit bland. But that's how it works. Because you can send anyone an email. But that's really not enough. You have to repeat it over and over.

mr.

So it is discussed and decided at different times.

a

Yes. But I do think that those decisions of "what do we choose?", we always do that in a group. So just like those brainstorming sessions at those lunch meetings, it's also a group. And then it is also said of well what is low hanging fruit that you can easily solve. Then let's do that. But then the follow-up after that if something is viscous.... What we do very much in the care chain, the things that are easy to implement, for which there is a lot of support, we choose those. Because that also has the best chance of success. Because apparently the interests are big enough there too.

mr.

Yes Yes.

a

And if only one person wants it…. That's what we do with those brainstorming sessions, isn't it? If only one person wants it then... Yes, you'll never succeed. So that's how we do it. So I personally find that decision-making process .... I have the idea that it is okay. There are always those who lead the way and those who lag far behind.

m

You always have.

a

It always is. Then you just have to work around that a bit.

mr.

No, exactly. And when a decision has been made to implement a certain project or an improvement. What kind of activities or steps are normally taken to implement that improvement in practice?

a

Well, then let's see what it takes? And who are all involved. And then we put those people together and then we turn them into a working group. And if necessary, you can expand a working group with, for example, someone from Lean. And they are... I must say we are very well supported by that department, huh. So people from Lean who support us very well. I think that's sensible too. Because they can analyze a problem and if it doesn't work, they can also address someone else about it. That's also good, more independent people than being approached by a surgeon.

m

Yes,

a

And those people understand. They have learned how to get support for it. So you have to include that. And we are also very well supported by ICT. Which is also very important. And the management. And we are also helped to write a business plan. If at some point everyone is involved. And everyone has had their say. Then we write a business plan if money is needed. And then you try to get it. And then the next step is implementation, huh? So first you look at what everyone in a core group is happy about what we're going for. Like back then with you and the dismissal process. Well, we've had many meetings about it, and then you're going to implement it. And you bring in the people who also have to implement it.

m

Yes.

a

And if necessary, we conduct a survey. And then we're going to implement it and then you have to continue it again. So it's important again, isn't it, that we're going to look at it in a while. How are things going? But we're now working on a way to get that CRP faster. And I've had several meetings with the clinical chemist about that. A lady from the lab, who is also a specialist, is also a nice profession by the way.

mr.

Okay

a

Then we try to prick it faster. But that, that is a very large comprehensive project. Where actually... Yes, the whole hospital wants to be pricked much faster. So you have to broaden that. Now let's go..

m

Certainly not the low hanging fruit.

a

No, definitely not the low hanging fruit. But what you can do, and I wasn't always like that, is put a device in the ward so that the nurses can do it themselves. And it turns out... I went to the children's ward, they have such a device. And that is really very easy. And that will mean that patients can go home faster because the CRP is known. Or that we know faster that the patient is deteriorating, then the scan must be made. And that is really an improvement process that I have really longed for for years. And because we have that care chain, you can really take steps. Do I have time to meet with her? A beautiful piece is being written. A business plan is made. And then we'll just order those things. And then the nurses are taught to be able to do that injection. So yeah, how beautiful is that, huh?

m

All those steps you just mentioned are mainly coordinated from the DB?

a

Yes, there is always someone from the DB who is the problem holder. And yes, I think Mandy and I are especially problem keepers of that sort of thing. And then me even more than Mandy. uhm. Because those difficult improvement processes are really part of the entire chain. And Mandy, of course, is more involved in the beginning. And Mandy is also actually more adopted to be a binding element. That's more her job. And I think I'm more into the improvement projects.

m

Okay.

a

And then you also have to be a bit above the parties. Mandy thinks that's all very exciting. And I don't find it all that exciting. I've done so many things already.

m

Yes.

a

Yes, so also within our DB we divide it a bit according to what people like, what suits them, huh.

m

You already mentioned a bit about support from the hospital and from the management. You also mentioned that there is support staff such as the man from Lean, ICT. So you are well supported in terms of personnel and resources to really implement improvements?

a

Yes, I think so, yes. It really is much much better than it used to be.

m

Yes. And also in terms of hospital management, who are also involved?

a

Yes. Because they are the boss of... Well, not the boss but they are involved in those care chains too. And then once in a while, once every few months, they attend a meeting of the DB. But yes, the funny thing is that precisely because it is guaranteed, it was conceived by the management, they want it to be a success, you are simply supported. So I don't have to speak to them every week at all. But you can just see from everything that it is an important item for the hospital. Those care pathways.

m

Great, then you don't have to think about that and you can look at the content.

a

Yes, you can work on the content. And you can actually take steps.

m

Yes. And if we look at quality improvement, in your experience, how is that valued by people in the healthcare chain, but also more broadly throughout the hospital? Is that something people find important?

a

Do you mean improvements in the healthcare chain?

m

Yes, quality improvement.

a

Yes Yes Yes. In fact, we are also judged on that these days, hey as specialists. Yes. Part of the money we get. How we get paid. Also checks whether you are working on quality. Happy finally, huh. Because in the past there was no control at all. And everyone could really do whatever they wanted. Yes, no that has improved a lot. Yes.

m

Do you also notice in the workplace that people also find it an important item?

a

The care chain and quality? Yes, because we did a survey. And then it seems like the people who really have the feeling of the care chain, I feel involved in that. But that is, those are all people in the healthcare chain. Look, surgeons are always afraid that the healthcare chain will decide something about them, aren't they? So yes, they could be a little suspicious at first. That's also about happiness. I said yes, that's all very marginal. And it's not about you at all or surgery times and stuff. They had to get used to that. But yes, the nurses and people who work in the lab or on the X-rays, they all think that's fine, they just let them all happen, don't they.

m

Well, fine. Yes. And if you look at the people within the healthcare chain. Do those people really have the desire and willingness to change if that improves quality?

a

Well, look, it's like everything, isn't it. There are always people who don't want to change and people who really want to. And yes, that's part of it. So uh. But in general I think the hospital is change-minded.

m

Yes.

a

And uh. Yes, that most... And whatever we just do. Very pragmatic, if people don't want to change, well then we don't talk to them. We only talk to people who want to change. So there are representatives for each department, who we call ambassadors. And they're all enthusiasts, aren't they? So you can say avoid those negative people. And yes, it is also from the management that they want it. So you will have to delegate someone at some point to improve. I don't see any resistance to that.

m

Okay, and if you look at improvement projects. Are they often people or colleagues who already know each other? Who will carry out such a project together? Or are they sometimes new people who don't know each other yet?

a

Sometimes people who don't know each other yet. Yes. There really is more connection. And that was the goal of the healthcare chain. And the aim of the care chain is therefore also that people find their work more enjoyable.

m

Yes. And also in terms of collaboration. Do you notice that the collaboration is going well?

a

Yes, that you talk to each other more and more, yes, if a unit is standing in front of that patient.

m

Yes. And these are often people who have previous experience with improvement projects or quality improvement. Or are they people who have never done that before, say?

a

I actually don't know. I can't answer that.

m

Okay,

a

You know me. I'm just going to rant very enthusiastically. I'm not going to ask have you ever done such an improvement project. Yes. In general, I do think that the people who are open to change will cooperate, don't you.

m

And such an improvement team also has the right skills. For example, to implement an intervention?

a

Yes. We're just looking for the people who, uh... Look, when people are enthusiastic, it becomes much easier anyway. And if they need help, they support them.

m

Okay, glad there's that support.

a

Yes.

m

And in terms of financial incentives are they there? Should people do this on their own time? Or are you compensated in terms of time or money for that sort of thing?

a

We are compensated in time

m

So the DB?

a

The DB is compensated. Yes, he will have time for it. And so is money. And the other people just have to see it... But they really only do projects that have to do it in their own time. But I do think if there were a problem and we go to the management and say 'wait a minute, this is necessary for that improvement plan', then you will get that too.

m

Yes. And in terms of data, there is also a good system with which you can collect the data you need. And can use for improved projects?

a

Yes, that is always very difficult with intestines. Because not everything is always kept digitally.. But we are supported, also by a data manager who sometimes also has data. You know that person. But you have to ask her things very specifically, she won't do things on her own. No, you have to get behind that. But yes, they... There is support, but yes... In other healthcare chains they are still very busy capturing everything in digital codes, but colorectal is so complex, we are not there yet.

m

So you do have data, but not always all the data you would like.

a

No, it is it anyway.. But if we would ask the ICT. Do you want to make that data a box? Then make them, then a few weeks later you have a booth. Only then do the doctors have to fill in the box, you know. I find the implementation the most difficult of all.

m

Yes. That is often difficult in a viscous organization such as a hospital.

a

Yes, that's really hard. And you're not the boss either, are you? I am not in charge of my colleagues. So otherwise I could just say yes you are dysfunctional. That is not possible. So uh. It also takes a bit of good will.

m

I only have a few questions left. When I look at clinical care. Have the improvements made so far really improved clinical care? Did that have an impact?

a

Yeah, because we get the catheter out earlier, huh? And also, what you did with that dismissal process. That's real, those are real improvements.

m

Yes. And are there certain factors that hinder an improved project or make it more difficult to implement?

a

Yes, because I told you. The hardest part is always when it costs money. And the implementation of that people are going to apply it. And we are just… for example, the nursing on the A3 is very well organized. So you can make changes quite well with them, but with doctors or physiotherapists it becomes much more difficult. J because of that relationship of authority, huh? We are not in charge of them.

m

Yes.

a

So it is.. You are actually the great inspiration. But you don't have any, I don't have any power or anything over them. Yes, that remains the better inspiration work. I also really like doing that.

m

Yes. And are there certain factors of which you say yes, that actually make it easier to implement an improvement?

a

Yes, that good support, eh, from the…. That you are not alone crying in the desert. But that a business plan is also written. That the people of Lean participate. That there is financial support if you ask for it. It is much better secured in the organization that the ICT people really provide good support. That saves everything.

m

Yes, of course. Well, then the last question. Is there anything else you would like to share with me. What do you think is important?

a

ummm have a look.

m

An important topic or something else we should talk about?

a

You mean about quality improvement?

m

Yes.

a

Well, I think it's important when you choose projects. That you choose a project that is promising. Because people are excited. And delegating is also important, if you sit down and do everything yourself then, yes, you get very busy. But the others are not involved. And then it becomes very difficult to implement after that.

m

Yes.

a

So I will really recommend if you talk to others about putting people to work. And then support them well, so that they will also wear it.

m

Yes, if you do everything yourself, you will eventually get a burnout. And then you can't do anything anymore.

a

If you have to do everything yourself, you have the burnout and people say what are you so worried about

m

Yes. Yes.

a

Yes. Then you have to let them do it themselves.

Interview #2

m

Then I'll start now. The first question is what role do you play in the improvement team?

E

Well, as a nurse specialist we often contribute improvements to the ward. And if we are also involved in the improvement. Then we usually have a leading role in that. But it depends a bit on what the improvement is and what is needed for it. Often we also send the nurse a little more to take care of things themselves.

m

Clear. Were you also at the lunch meetings and the process analysis?

E

I have never been to the lunch meetings before. But once at the end of the day. Such a feedback thing.

m

No, because then we had the entire healthcare chain. All intermediate steps mapped out. And that was interesting. So I wanted to ask about that. But if you weren't there, that question no longer applies.

Okay. The decision-making within the healthcare chain, and I mean in particular decisions about whether or not to introduce a certain improvement, so to speak.

E

Yes.

m

How do you think that is going at the moment?

E

hmm. I think that's a good question. I think that's going very well now… uh at least, of course I can only judge about the clinical part, and I think that actually goes through the management team of the department.

And if things need to be improved and ideas are ahead. Then I think that this is mainly discussed with them and decided whether it will actually be implemented.

m

J. By the management team of the department, do you mean Evelien for example?

E

Yes, or the team leaders who work under her.

m

Yes, you do not mean so much looking at the day-to-day management of healthcare.

E

No, in my experience not necessarily about the clinical bit. No.

m

Yes, okay. And what do you think of the current way of decision-making. Is that going well? Can it be better?

E

Well, I think it's not necessarily a wide range of care now. I think that if a point for improvement is seen in the department and, for example, a student starts working with a graduation project. Then I don't think it is necessarily something that is known within the healthcare chain at all. If you're talking about somewhat larger projects, such as those that have now been taken up in the healthcare chain, I think they are involved. And that, of course, came up once in such an afternoon meeting that the healthcare chain is then briefly linked back. But I don't know how that decision-making goes.

m

Okay. Yes, and if I understand correctly, would you rather broaden it a bit more in the healthcare chain?

E

Yes, I have the idea that a lot more is happening than is per se known in the healthcare chain. But I don't fully understand that.

m

Okay. Then about implementing improvements. What activities are undertaken to actually implement a particular intervention?

E

As in who that..?

m

I just mean it very broadly, because that depends of course on what you are going to change. Yes, but a little in general. What kind of activities are undertaken to improve something in the department for example?

E

Well, I think you mean, for example, literature research or that sort of thing. Or not?

m

among other things, but also more yes, we have a nice idea. But hey, who's going to do that? And sometimes that lingers a bit, so to speak.

E

Yes, exactly. Well, on the ward we have nurses in charge who are also in a focus area. If there are things that need to be improved, this often goes through the group of attention fielders from that area. And they communicate that within the rest of the team. And uh. Yes. What about activities? I think that. Yeah, I don't actually know.

m

Okay clear. For example, if an improvement is made. How are the people involved informed? Tell me. Or included in it?

E

Well, basically I think the smaller things through the mail. And of course there was also such a meeting a while ago, in the afternoon with feedback. An update of innovations or improvements. So I think it will also be told across the healthcare chain.

m

Yes, clearly. And further, as you just said, there is a certain direction, so to speak. In terms of things being done. This is divided according to focus areas.

E

Yes.

m

Okay. Yes, clearly. Then some questions that are more about support from the hospital. uhm. Is there support from the hospital for improvement projects?

E

Yes, it's definitely there. But I have no personal experience with that.

m

Okay.Yes. Then the next question is also less relevant. Because I wanted to ask whether concrete resources such as money, personnel or training are available?

E

Of course you have. I don't know what that's called. That you can win a grant for your research. For example, or improved trajectory. Then the antonius Academy gives money to do that. And yes for the rest Uh, I don't know.

m

Yes. So if, for example, you would now like to implement an improvement. Then you basically just have to do that in your own time?

E

I do that in my own time. Yes.

m

Yes. Okay. And yes, a little bit of what goes on here. The hospital board or more senior management. Do you feel that they are involved in the improvement projects?

E

Yes. I think it depends a bit on the size of the improvement project. In the department, I don't think we are so much concerned with management. I don't actually think so.

m

Yes, okay and quality improvement in the hospital and in your own team, what is the culture around that? Is that very much appreciated, is that appreciated? Or are there other thoughts on that?

E

The culture? I didn't understand you very well.

m

What is the culture around improvement initiatives in the hospital and in your own team?

E

I actually think that's a really good thing. And very active. I think everyone is constantly looking for uh we're doing well. And what can be improved. And even if you do indeed come up with a plan to improve healthcare, for example. I also think there is quite a bit of support for that within the team. And that we do that. Yes.

m

Yes. It is supported, say from the staff?

E

Yes. I think so. If you can at least substantiate why you changed something. And what will get better.

m

Yes. With that you have also somewhat answered the next question that I wanted to ask, because the desire and say the willingness to change is there?

E

Yes, I generally think so. Especially within the nursing team. But then, huh. That you can substantiate why you want to do certain things differently. Because changes often cost quite a bit of time and some investment from, among others, the nursing team. And if you don't substantiate that well, I think they don't always want to cooperate. And if you include them in your process. I think there is a lot of support for that.

m

Yes. Yes. Good reasoning is always important.

E

Yes. Yes.

m

And then if we look at the people involved in improvement projects, are they colleagues who already know each other? Or are those people who then come together but have not met each other before?

E

I think that varies a bit in terms of what projects you're working on of course. The projects I see in the department are often colleagues who know each other.

m

Okay. And do they have previous experience with improvement projects?

E

Yes. Well, in the healthcare chain I don't know. I think he does, for example, as a board member. And I recently did an improved project myself and presented it within the healthcare chain. So yes, I think.

m

Yes. Okay. And how would you describe the collaboration?

E

as within the healthcare chain?

m

Yes.

E

Yes. I think uh. Well, I think very well. And I think everyone has the same goal too. But I do think that it is still very islands and that I am not. I don't hear what's going on, for example, because the healthcare chain is of course very large. You completely figured that out. I do not hear what is happening or being done in other parts of the healthcare chain.

m

Yes, okay.

E

I think the collaboration itself is there. But I have that, I don't have that insight.

m

Yes. So that they are still a bit too many islands on islands.

E

Precisely. Yes, I still think that. We're just getting started, of course. But I think it can still be expanded a bit more. So that you are more one.

m

Yes. And if you look at the improvement team. Are the right skills also present? To implement interventions and improvements?

E

Who is on the improvement team?

m

Yes, that also varies a bit depending on what you're doing. But yes, in general, if you look like that in the care looked. Or if you look at your experiences you've had.

E

Yes. I think it's just a very mixed group. So I think you certainly have enough experience in that.

m

Yes. Okay. There were also financial incentives. Were people compensated for the time or effort they put into it? You just said that you basically do everything in your spare time.

E

Yes. Well, I do in between during my work, you know. But yeah, I don't know if people get compensation for that, if they do an improvement project. I don't actually know.

m

Okay. Yes. If we look at the data, is there also a system in place to collect and deliver data?

E

I think so. I don't know what it's called anymore. That's something we've talked about. That is..

m

Do you mean the dashboard?

E

No, I don't know if that's another. Maybe a data system that you've done other studies with. That is also possible. I don't have, for example the improvement project I did, I didn't deliver it somewhere or something to the healthcare chain that they can store it somewhere. No.

m

Okay clear. And interventions in which you have been involved or the improvements in which you have been involved. What impact did they have on clinical care?

E

Well, that's actually quite a lot. I looked at the home care needs of patients who receive a stoma. And previously, 95 percent of home care patients went home. And now, and I am no longer active in that project, but the last measurements we did were at fifteen percent. So I think people are much more self-reliant and less home care is needed. And therefore less healthcare costs in that regard. In the clinic, this did not necessarily mean that there was a lot more work for nurses. Precisely less because patients were more independent.

m

Yes, so quite a big impact when I hear it like that.

E

Yes. Yes, that's nice.

m

Were there also unexpected effects of an improvement? Did you just mention that the workload didn't increase?

E

Let me think.. I think the cooperation between the stoma care nurse and the department has really improved, indeed. And well, we didn't expect it to be such a good result anyway.

m

Yes. So as a side effect actually better cooperation between the specialists involved.?

E

Yes.

m

Yes, those were actually my questions. Finally, you wanted to ask. Is there anything else you want to give me? Or something I should have asked that you think is relevant?

E

Well, I was. That is more common across the healthcare chain. Was I ever thinking of hey how, how do I look at it now and what I do, what struck me during that meeting when I was there. Is that it is especially very focused on the surgical patient. And I think that if we naturally have a healthcare chain, that may also be due to the composition of the board, I think that if you really want a healthcare chain to have colon cancer. That there are always the non-surgical parts, is also important to discuss.

m

Yes.

E

And yes, that also made me think during that meeting of well, the content of this discussion is not very helpful to me. Hey because I work in the clinic and we talk about a lot of things in the clinic. So I would find it really interesting. To learn or hear about processes before and after the patients are in the clinic.

m

Yes. Yes. I think that's a very good point you bring up. Yes. Which probably also increases involvement in the care chain. If people feel that that is also relevant for them.

E

Precisely. Yes. I am also curious about the patients who just before or around the diagnosis, how is everything going there and what kinds of things are being improved there? And also the part when we refer patients to, for example, the oncologist. And what are the processes there? And how? What's happening there? I think that would be really nice to know.

m

Yes. So you don't want to stay alone on your own island. But also look at the neighbors.

E

And I think uh. Last time it was always outside working hours. And we try to approach it a little bit that we can arrange things within working hours. Because you can meet every day until ten o'clock in the evening if you want to. And so that was also something I thought yes, if we want to improve that properly or get more support across the healthcare chain. Then it might be good not to always schedule it after working hours.

m

Yes, of course. Good point. Then I want to close this.

Interview #3

m

The first question. What role do you play in the improvement team in the care chain?

E

in improving the care chain?

m

Yes, exactly.

E

Yes. And from Uhm. From the perspective of radiology, I am the point of contact for the healthcare chain. uhm. Well, they started not so long ago of course. And the agreement is kind of that if there are things that can be improved or that are working, that we. that uh. That, for example, he or someone else, if they can come to me and that I, that we then see how we can solve that.

m

Yes,

E

to date, that has not actually happened yet.

m

Okay. You coordinate from radiology.

E

Yes

m

I think you've been to those lunch meetings. However?

E

Yes. I've been twice I think. And yesterday or the day before yesterday, I think it was another one. Only then I had another meeting at the same time and then I couldn't join

m

And the lunch meetings, where the process analysis is done, as it is called, what did you think of that? Did you find it useful to do?

E

uhm. I don't know if I was there at that trial analysis. Because I don't really remember any of that.

m

Yes, that was a while ago. But that meant that they covered the entire healthcare chain, from the very beginning that the patient is presented to the aftercare. All these different steps have been mapped out.

E

Yes, just a kind of patient journey.

m

Yes. And that each step is asked, well okay, what could be done better here in your opinion?

E

Yes,

m

Does that make it a bit clearer for you?

E

Yes. No, then at least I understand what it is. But yes, I can.. I don't know if I was there. Because I can't really, uh, remember hearing anything about that. How did those steps go in radiology and what could possibly need to be improved there?

m

I'll take a look, then this question doesn't apply. Then we move on to the next one. You said you're a point of contact.

E

Yes from the beginning, the healthcare chain has only recently started.

m

beats. Yes and have certain activities been undertaken to implement interventions. As far as you know?

E

Yes for patients who need to get certain tests. Yes, but that's something that's always been going on. So why. So uh. Patients should simply be able to get an examination at short notice. So there are just places reserved for that. And I know a few weeks ago that there were some problems. That there were too few places. But then more places were made available again. And I think that has now been resolved. uhm. Yes. So uh. No. In my opinion, uh in terms of radiology is not really that much yet. Yes. Not really much happened yet. I haven't been needed very much yet.

m

And before the healthcare chain started, some things had already been done. Or not then.

E

uhm. Well, I think everything was going the way we wanted it back then. So.

m

And in terms of support from the hospital. Do you have the idea that there are really resources available. If you would like to improve something? That frees up time or money? Or is that support staff, if you want to implement an improvement project?

E

uhm. Well, I'm sure there will be. But I didn't really notice that. But yeah. Well, again, we haven't really needed that yet either. There has not yet been a question from that care chain, at least to me, that we would like to change this.

m

Yes.

E

So yes. It's a bit in that respect, I think a boring interview

m

Oh, no I have other questions that are also relevant in themselves. Okay. For example Umm. Yes. Quality improvement. How do you think that is valued in the hospital and also specifically in your own department? How do people look at it?

E

Well, we are constantly working on quality improvement. For example, I acted as a point of contact for the bowel cancer care chain. Another example is the prostate care chain. I am also involved in that. uhm. Yes, there are those meetings too. And from time to time things come up like well, this is what we would like to do. Can you adjust that? And then we implement that. Now we have recently added something to the standard report. What they wanted in there. And we have now implemented that. So at least that's now, it's running now. And that is at least something that improves the quality. And yes there are, actually in all areas there are daily things that we do and adjust to improve quality. So that's something. Just an ongoing process. Not specifically for the colorectal cancer care chain. But that's something we're just working on.

m

Yes, so you are constantly working on it. I think it is also seen as part of your work

E

Yes, of course. Yes. Yes. We are, of course, supportive and service-oriented. So yes. We try to do the best we can for our applicants.

m

Yes, okay, clear. And. if you look at yourself or colleagues, do they really have a desire and willingness to change their way of working if it improves the quality?

E

Yes, of course. Yes, of course. Yes, there is. Well, within certain limits. Because of course we sometimes run into problems, against the limits of what is possible. Of course we don't just work for the surgeon. But for all specialists in the hospital. And everyone wants to change things like more MDOs or certain things. Or a fixed point of contact or priority when requesting diagnostics. Yes. So yes, we sometimes run into the limits of our abilities. However, we have also had a certain consultation for a year now. Which we have twice a week. In which we look at the waiting lists where the bottlenecks are. Where can we deploy additional programs to do something about this? Yes, also something that improves the quality. uhm. But yeah, sometimes it just is. Yes, that's what I meant so sometimes we don't have more time than we have.

m

You don't have infinite possibilities of doing things.

E

No no. No, for example. For example, there is now talk of perhaps starting a new MDO for T1 tumors specifically. Yes, but yes, that is again an extra MDO. And I mean those MDOs that are very useful and important as far as we are concerned. Only then should you have a radiologist who will prepare the MDO. And in the time he prepares that and goes there he can't beat scans. So uh. Yes. So sometimes we run into that.

m

Yes.

E

uhm. It is difficult to find a good balance in that.

m

Yes. And if you look at your quality improvement activities. And that doesn't necessarily have to be just for colon cancer. But also more in general.

Is that mainly within the radiology department? Or does it sometimes involve collaboration with other departments? That you implement an improvement together?

E

The same goes for other departments. For example, last year we also carried out an improvement project from the hospital. We looked at patients who had been helped in the emergency room and for whom an ultrasound had to be done. So we went with some of those patients. From the beginning he came to the A&E to the radiology department. And then went back again. Then we looked at what are the bottlenecks there, what could be improved? How can we improve quality for that patient? So in the end, together with the emergency room, we drew up all kinds of points for improvement. And we've been doing that slowly over the past few years and over the next few years, we're implementing that wherever possible. Yes. The same applies to patients who came from the GP. For an X-ray, we did the same for it. uhm. Yes. And of course we also work a lot with nuclear medicine. So we try to work together in that area as well.

m

Are these often colleagues who already know each other? Or is that that through such an improvement project you qualify with colleagues that you didn't really know well before?

E

Yes, both. Both

m

both. Yes. Yes. Okay. And if we look at the skills to implement an intervention. And I mean it very broadly. Do you think the right skills are available in the care chain or in your own team to really implement improvement projects?

E

Uhm yes, I think so, I think those skills are there. But I also think that uh. That again sometimes, maybe, yes, the time and the pressure that is already there of the day, shall I say that sometimes those maybe, yes, can cause uh that certain things are implemented less quickly than possible would be. Yes, then what you would like.

m

Yes, due to a lack of time and manpower?

E

Yes, exactly. So you are actually a bit limited by the resources you have and especially, for example, by time and manpower.

m

Yes,

E

precisely. Time. Yes.

m

uhm. And how would you describe the collaboration within your own department or with another department? If you are working on such an improvement project, does the collaboration go well or could it be better?

E

uhm. Yes, I think it's going well. Yes, I am too. I'm also the department's medical manager, aren't I? So I had that sort of thing. I am often involved. um. The only thing I occasionally run into is the lack of time. But the collaboration goes further. That's fine. Yes.

m

Okay. And previous experience with improvement projects. Is that also present? Or is it sometimes completely new to certain people?

E

No, it is present in humans. That's not new.

m

Okay. And are there financial incentives? Are people compensated for the time and effort they put into it?

E

Uhm not specifically for the care projects. That's more. um. Look, the quality is just part of my portfolio too. As a medical manager. uhm. So basically I get paid by the MSB one day a week. So um. Yes, there I will be. That day I get compensated for that sort of thing. But there are also many other projects that have to be done on that day as well. This does not mean that we will receive any specific compensation. We still have two quality advisors in the radiology department. And they are also financially compensated for that.

m

Yes. And in terms of data infrastructure, there is a system available with which you can supply data. For example, for quality improvements?

E

uhm. Well, there isn't a specific system available for that as far as I know. Of course we have that quality, annual report every year, don't we? But two radiologists from my partnership do that together with the partnership secretary. But yes, there is no specific system for that.

m

If you look at certain improvement projects that have already been implemented in the past. What impact has that had on clinical care?

E

erm. A good question. Well, if I look at that project we did with the emergency room, for example. uhm. We have analyzed that entire patient journey. And we have. um. seen certain things. For example, time is lost. Whether things are unclear to the patient. Those were things that uhm that were relatively easy to adjust. So at least I think that has resulted in patient satisfaction and time savings. Yes, the same applies, for example, to the prostate care chain. Yes, we have helped to set up a sort of yes real-time, just like with the colon cancer care chain that is also there, I believe, the dashboard. So yes, I think that also provides quality and insights.

m

Yes.

E

uhm. Yes. So things like that.

m

Yes. And if you then want to implement a project that is better for improvement, are there certain factors that facilitate or counteract the intervention?

E

uhm. Difficult question. Yes, but uh what always works is that you just have a good relationship and cooperation with those colleagues and short lines of communication. Yes. I'll just say that you also have something for each other. So that.

m

So there is a culture within the department that you support each other with that sort of thing. That you do, yes, try to improve quality together.

E

Yes, of course.

m

And then perhaps in terms of factors that make implementation difficult, is that perhaps a limitation in terms of time and manpower?

E

Yes,

m

Okay. And do you also find that the workload increases with certain interventions?

E

uhm. Well, I don't really have concrete examples of that. But there is potential of course. If, for example, there is a demand for something specific from a healthcare chain. Yes, For example, faster reporting or extensive reporting. Yes, that could potentially involve more work. But I don't really have a concrete example of that right now.

m

Yes.

E

And certain data that must be supplied, structurally. Yes, that could involve more work.

m

Yes. And then a question about decision making. In your experience, how does the decision-making process go about choosing and implementing improvement processes. Is that something that is organic or is it preceded by a structured process?

E

I really have no idea. No. uhm. Yes, I think it's going quite organically because uh.. Well, of course you now have that healthcare chain. And then you have a number of people who are affiliated with that. And everyone can simply make their own voice heard and participate in the decision-making process. I think that is fairly democratic and organic.

m

Yes. Okay clear. Then the last question is there anything else I should have asked you or something you want to share with me that you think is important?

E

uhm. Well, I know that, for example, there is a dashboard in the prostate chain and there is also a dashboard for colon cancer. I just don't think that's really accessible to us. So I don't really know how I could access that as well. Because I think there are interesting things to take from that and possibly to add. So I would like to have more insight into that.

m

to be able to improve the quality even more, say?

E

Yes, exactly. if you have insight into your data, then you might also be able to see where you could send. Or things could change. So I would find that interesting, for example. I do not know whether this can be seen in real time, for example, how long patients have to wait for certain examinations. That would be very interesting to me.

m

That is therefore a good improvement for the future.

E

Yes.

m

Yes. Okay. Well I'm through with all my questions. Okay. Well, thanks for the time and your answers

E

good luck.

Interview #4

m

What role do you play in the improvement team?

huh

I support the day-to-day management from my work. So that's Lean.. from a Lean background.

Yes, I support db. So I sit, I also participate in DB weekly. And I help them with all kinds of things.

m

And so, among other things. Such as organizing and setting up those lunch meetings and the process analysis.

huh

Yes, we started that last year. Yes, we have made all kinds of process analyzes for the summer of 2020. So the entire healthcare chain has been mapped out. Finally worked out on a large sheet of paper.

And we have around the summer period. Have organized five lunch meeting with yes, the ambassador from the chain to watch. Is this the process? Yes. And what goes well in the process? In the overall process. But also in your part piece. And we especially looked at points for improvement from the chain. Yes, that came from those lunch meetings.

m

Yes. Precisely.

And what do you think of the process analysis?

huh

uhm. Can you ask your question specifically? Because what do you mean.

m

Do you find it useful to actually do this as standard in the care chains? For example now with colon cancer, but would you actually want to add it as standard in all healthcare chains?

huh

Yes, I think so. But that's a lot of work anyway.

m

Yes,

huh

For example, we are working on MDL piece with the MDL outpatient clinic. To look at what, how does the process work. But at least there are some gaps. But then for me the basis is often to make the process analysis. To understand the process together. And often in his process analysis it often comes to the fore that people do not know exactly what is happening or gain more understanding of each other's work. And based on the steps that are available, you can still look at okay how is the step going and what improvement can we implement. So I think that is a very useful tool to use at the start.

m

Yes. And once the trajectory is running, it is something that you would also do periodically. Or are you saying that's just a one-time thing. And then it's done, so to speak.

huh

Well, actually not. The best thing is actually that you map out a kind of the current picture. So map the current process. That you actually eventually want to say okay we want to improve the process and that you periodically go through that again to see if the process is still running

As it should be. And if improvements have been made, you can also adjust the process on paper.

m

Yes. Yes.

huh

But for me it's always a living thing that you say okay we now have for example… Today we had a conversation. We have identified areas for improvement. And after x number of months you will look again from ok. Now what is the persecution? What are the next steps or other points for improvement that you can implement in the process.

m

Yes, exactly. Yes.

huh

I mean you take the most important now. You implement it and then you apply it and then you look. Well, the process is now running better or not better. And then you pick up other points for improvement.

m

Yes, exactly. That is really a kind of feedback loop that you always have.

huh

Yes. Yes. beats.

m

In the current working method, do you think that process analysis is feasible?

huh

Is it feasible in the current method?

m

Yes. So, uh, do the healthcare chains really need to adapt their way of working in order to be able to carry out that process analysis?

huh

No, they had to have time to discuss it together. That is the most important.

m

Yes. In fact, time is the most important limiting factor, so to speak.

huh

Yes, but in itself it is also relative, isn't it. So, for example, the lunch meetings that last an hour and he and I did a lot of preliminary work by working out part of what we knew and testing it. We have also had a number of sessions in the preparations. For example with MDL doctors. For example, to work out a piece. Or uh also with the palliative trajectory. And that takes one and a half, two hours for a person to think along with us about how the process works.

m

Yes, So it is made as easy as possible for everyone to be there, so to speak.

huh

Yes, some preliminary work done. That things have already been worked out. And then with some we didn't have that foreknowledge. And we're just really off. Well, started from scratch, just pasted yellows and mapped the process together.

m

Yes, exactly.

And would you like to change something about the process analysis as it is currently done?

huh

Well, the way we did it back then, I think it was well done. Of course very much from, yes, more gut feeling, huh? So the lunch meetings were also okay. What's going well. What's not going well? Simply also about a bit of collaboration or about patient experience. What we didn't do at the time was simply process the data in it. For example about lead times or waiting times or about error percentages and going home. We will be working on that shortly. So I think it's really cool to connect the existing process to data.

m

So that's something you might want to add to the process analysis. Yes, okay.

huh

But. Yes, of course much more. Then you can substantiate many more things. So I'll just name what there are ten steps in. And those steps take so much time. Or there is a lot of waiting time, then you also have a conversation with each other about how come? That it takes so long. Or that patient has to wait that long. And then you go based on the facts. Gonna improve even more.

m

Yes, some more support for your theories and hypotheses that you have.

huh

Yes. That's right.

m

Okay clear

And then a few more questions about decision making.

What do you think of the way of decision making as it is currently done to select improvements?

huh

Well, that now mainly lies with 1.. well not decision-making, in the end everyone is autonomous in the chain to make decisions about what they will and will not tackle. It's more for me. A bit of control and see what the results are. That there could be a little more attention.

m

Yes, that's crazy of course. It's changed a bit now. Because we used to have an improvement team. And now you have a daily board. So decision-making is somewhat more concentrated in a smaller group of people. Yes. And yes, that also has its pros and cons. You can make decisions faster because you are with a smaller group.

huh

Yes and you are on such a daily board, everyone has their own point of attention. And yes, for the process improvement, Mandy has mainly been involved in this, together with me. And yes, that just takes a lot of time. So I think what we could have done differently and still can do. Is that you get more areas of attention for certain chunks of improvement. So you can really cluster some points for improvement and say who is the point of contact for such a cluster. Because then you can easily invite those people. For example, in a DB. To see how things are going and progress.

m

Yes. So in decision making. Is that something we could possibly improve on? Just say or change?

huh

Yes,

m

Okay. What activities were undertaken to implement improvement interventions?

huh

Well, those were the lunch meetings. But also invite people to DB. There are, of course, all sorts of discussions. But of course that is all still very much in the yes. In your own field, say to see what can be improved. And it's not a consultation across the entire chain. I do think strongly of those lunch meetings to also repeat it to see we did it last year. And where are we now and where do you see more opportunities for improvement?

m

Yes.

huh

And I think it's good to repeat this periodically.

m

Yes, exactly.

And if you look at all those activities you just mentioned.

How did they come about? Was that a bit organic? Or was that really a very structured process?

huh

No, it did come about organically.

m

Okay.

And in terms of involvement. You just indicated that that in itself was, say, within the entire healthcare chain. But that it still remained somewhat within its own department.

huh

Yes that's right. Of course everyone has their own process. Yes, there are still islands in between. So things are getting better, I do notice that more consultations are being done. And that could be even better. So you want to look a little more beyond the borders. If you are partly responsible and then see what comes before my process? And what comes after my trial? That it could be even more integrated.

m

Yes, clearly.

And how are all involved professionals informed? During a certain improvement project, so to speak. Or during an improvement they are working on.

huh

Well the DB organized a webinar in December to inform people about the developments. And they publish a periodic newsletter.

Well, two two was another medical consultation. So certain topics from the chain are also treated. Only then will you reach a certain limit in the chain. Not the entire chain is included. And the question is whether that is always relevant. I don't think so.

m

No, exactly Right. You can't sit down with thirty or forty people.

huh

No. No. Such a webinar works fine. Because then you have, all kinds of pieces from the chain were explained there by someone from the chain, an ambassador. So that. That is very beautiful.

m

Yes. Okay, yes.

And now some more general questions about the hospital and the environment in which the improvements took place.

How has the hospital supported the project?

huh

hmm, that's a very broad question.

m

I can narrow it down a bit more.

Were the resources actually available? Then I'm talking about concrete things. Such as time, money, staff, courses. Those kind of things.

huh

Yes, that's for sure. A budget is also exempted for the healthcare chain. So where people could do all kinds of things and time is set aside for the DB itself. Who were given four hours a week to do DB tasks.

They have received support from us and at least from Lean. So Lean and VBHC.

And I was also able to spend time with BI. There was the necessary attention by, for example, Paul and Maurice, head of Lean and care logistics, who had regular contact with them. And Luke has been a few times. And periodically, of course, meetings together with other DBs, other care chains. Also had training in the beginning, also together with HR

m

Yes.

huh

The only thing we've had little or no support from is marketing and communication. And DB itself has a problem with that. Yes. Especially because we also wanted to map a patient flow. A patient journey. Always able to do a little more communication. Promotion, so we actually had to do that ourselves. Yes, sometimes that means staying. lying down.

m

Oh okay. Well, that might be an area for improvement than from support.

huh

Yes, of course.

m

Was the hospital board actually involved in this project?

huh

Well, as far as assessment came. Because Luuk was present at the discussion of DB. And some good conversations with him too. Anke also had direct contact with him. So that was also easily accessible. As far as I can judge.

m

Because he also has the care chains as a point of attention. I think.

huh

Yes, I think there is a subdivision there. I think he has colon cancer. I think there is a division in that.

m

Oh yes, okay.

How is quality improvement or VBHC generally appreciated in the hospital and in your own team?

16:42

uhm. Yes, that last one. The first is difficult for me to answer. uhm. Because how is it valued in the hospital? Yes, I think of course much better teams have started or are already running. And

so I think that's more and more embedded. Also the hospital and much more fame, so to speak. And from our own team. Yes. This year you were able to experience that in the Colon Cancer Care Chain. To work with both VBHC and Lean.

m

Yes,

huh

Unfortunately, I am dealing with someone who has naturally become ill and passed away. So that has been quiet for a while.

m

Yes. Yes, I understand.

But if I understand correctly. Are you actually saying that there is in itself a culture within the hospital in which… which in itself is positive against improvement initiatives and also supports that, so to speak.

huh

Yes. Yes, especially because that is also based on data. And yes, it is indeed alive. I think for me the term VBHC, still occasionally difficult for some people. How academic. The same with my Lean profession, if you talk about continuous improvement then that is much more manageable so to speak. Now VBHC is also continuous improvement. I can see that it is increasingly integrating with each other.

m

Yes, so you say quality improvement or improvement projects. This is well known in the hospital.

huh

Yes, that's right, do that by means of data, so to speak. That's VBHC.

m

And has the improvement team. Also the desire and willingness to change. And does the medical leader really facilitate or encourage this?

huh

Um the medical director. Yes I. Yes, I do find it difficult. I think the DB could play an even more prominent role in the chain. Because you're talking about improvement team now, aren't you, you're not talking about the chain? The healthcare chain?

m

That's right. Yes, in itself, that improvement team does not actually exist. Because now you really only have the DB. You could also apply the question to just the healthcare chain, so to speak. In general

huh

hmm hm

m

You said the DB could play a bigger role, you said. In what sense do you mean that?

huh

Well visibility and I think a little more steering on results. But I think that has also been a bit inherent, also due to the disappearance of the person who has become ill, that we have also paid less attention to that.

m

Yes,

huh

because it's not in the DB's genes, so to speak. To be very specific with data. Anke, of course, from her own field of expertise. But not everyone is very concerned about that. And that is the control that I think you need, also from VBHC. To get started with that data. Also show people what it is possible and what it is, so to speak. And of course we have already missed that for bowel cancer due to the loss of Jos.

m

Yes indeed. Yes.

And if you look a little bit broader at the healthcare chain. Do you think there is a real desire and willingness among people to change? For example, to adjust a working method?

huh

Yes, but I think it's kind of in the genes of the staff. The hospital staff, of course. Of course they always want for others. If it benefits the patients of their own working method. So that willingness to change is there, yes.

m

Okay. And the colleagues who were involved in improvements in the healthcare chain, did they already know each other? Or were they often strangers to each other?

huh

Well, everyone has gotten to know each other better through the healthcare chain. So what used to be more islands, so where everyone worked in their own field. But that we have already looked a bit beyond the borders of the islands. I also saw this during the lunch meetings. That people knew how to find each other. And that after that some better initiatives were started themselves. That people have looked up to each other. So that's nice to see.

m

Yes. Yes. Sure

huh

And through communication and a newsletter and webinar, it is easier for people to know each other. I'm going to talk or call or make an appointment with them. That it has been accelerated.

m

Yes. So that surely comes on the radar of people who they can reach for certain things.

huh

Yes. Through communication. What happened in the chain? So a webinar where an MDL doctor tells you whether someone tells you something later in the process. That people are interested in that. And so it is easier to find each other.

m

Yes, exactly.

And had the nurse, specialist or anyone else who participated in those improvement projects,

already had previous experience with improvement projects. Or was it completely new to them?

huh

Yeah, that's a really broad question, of course, isn't it? So not that they were very concrete, I think, busy with uhm .. Yes, I think there was also a colon cancer improvement team?

m

Yes, of course. Correct.

huh

So of course they are also used to working with that.

m

Yes. Yes. I think it's a bit part of the hospital for people. Because we have been working on improvements for a number of years. So that this is not necessarily something very new for most people in the healthcare chain.

huh

No, that's not news indeed. but what I do notice, of course, which we already did, also from my field of expertise, that people solve problems much more easily, but do not solve problems in a structured way. So yes, put out your fire, so to speak.

m

Yes. Yes there. That is why we are here to add some more structure.

huh

Yes.

m

And how would you describe the collaboration within the healthcare chain? Or within improvement teams that then take on a specific project?

huh

uhm. Yes, I think good. Can always be better. Well, that's kind of my area of ​​expertise as well. And that's my own uhm critical attitude, think of it can always be better, you know. So I also look at the colorectal cancer care chain. Consider that given the circumstances that really a lot has happened. But I also think so. Yes, of course it can always be better. But I think maybe we shouldn't go too fast either. You can expect some things to go very quickly. But..

m

ok, yes. So then you say in terms of cooperation that went well.

huh

Yes.

m

Okay, yes. And if we look at the skills, did the improvement team have, and then I also use the word improvement team a bit as an analogue for the care chain, did the people have the right skills to also carry out an improvement intervention?

huh

The answer is pending. Yes, I think it could be better.

m

Do you think there is a certain skills gap among the staff. What could help them make improvements?

huh

Well, there's more to it. That I think a problem will be solved. But all ad hoc, so to speak. So a problem is solved immediately. But I think it could also be approached a little more structured. To look more at, yes what are the causes of the problem. How can we permanently change that and continuously improve it?

m

Yes. So that, for example, if there are certain handles for this so that people can really tackle this in a structured way.

huh

Yes. Look from my field of expertise I also provide training, for example in the field of Kaizen, which is a very structured way to tackle a problem. Where you first also look at the real cause of a problem without thinking in terms of a solution.

m

Yes,

huh

that is also somewhat inherent in the work of many nurses or people from the care sector. Then there is a problem and it must be solved. Uhm so. Well, good. Then that would. There would be something more. More attention could be given to that. Yes. And what I do see. There is also the example from other healthcare chains, such as Trauma. They've already been to Seattle once. It was actually the goal for us to go to Kaas because then more of a common language is spoken.

m

Yes and that was done digitally in the end. But it's still better if you're physically there with the improvement team.

huh

beats.

a. It is not a must for me to all go to Seattle. We can also, so to speak, go to a reputable company around the corner, so to speak. But if you can speak the same language a little more, that's very cool. Because then energy is released again in such a chain.

m

Yes. So that could be improved in terms of skills, so to speak.

huh

What do you say

m

So in terms of skills of the team members, for example knowledge about Kaizen or speaking the same language, that's another thing that would be valuable.

huh

Yes. Well. In any case, a structured way of solving problems. But also just make sure there is a follow up. So we have agreed on this, what are the follow-up actions and how do we provide guidance. And how are we going to guarantee? That actions are also carried out. And that they are permanent.

m

Making concrete, monitoring progress. And yes things like that. Yes. Okay.

huh

Yes,

m

And then in terms of compensation, there are financial incentives for team members. For the time and effort they put into it?

huh

Not as far as I know.

m

Okay.

And is there a system available with which the required data can be supplied? In need of improvement projects?

huh

Yes, a dashboard has been built. And so a care path, let's say with data. Someone is working on that.

And soon, yes, I actually want to involve more specialists in the DB. For example, once a month. That she comes to go through the data as well. Yes,

m

so actually there is already some kind of data infrastructure in the context of the Santeon improvement projects as well.

Data analysts and the like.

huh

Yes, but well, that has also been left behind by the disappearance of the one who has become ill. And not directly a replacement for him.

m

Yes. He played a big part. Indeed.

huh

Yes.

m

If you look at improvement interventions, they have been implemented. What impact has that had on clinical care?

huh

Yes. I don't have them ready. But there are quite a few… um. Certain things are really well done. Like with stoma care, that obstruction protocol with prehabilitation, so to speak. So those are things that have gotten a boost again, I think. Also because of the care chain.

m

Yes. And is it really monitored? Or improve certain outcomes through an intervention?

huh

I didn't get that. And I haven't heard that in the DB either.

m

Okay,

So there isn't really a direct feedback per se from we're doing something, we're looking at what outcomes are affected by that? Or what really is the effect of the improvement, which is not necessarily kept up to date?

huh

No. Don't know with me. So it can happen, but not that I know of.

m

No, clearly.

And why there are also unexpected effects/side effects of implementing improvements. As far as you know?

huh

Side effects… Well, I don't know, Yes. Maybe people, which I do hear. That it is easier for people to find each other now. And make things easier to discuss.

m

Yeah, that's a positive side effect, really?

huh

Yes.

m

And, for example, in terms of workload? For example, has there been an improvement that increased the workload?

huh

Well, can't judge that.

m

Okay,

Were there certain factors that facilitated or hindered the implementation of an improvement in practice?

huh

Well, I guess what we were just talking about. More control over progress. That that could have been better.

m

Yes.

huh

And that makes it easier to see results.

m

Yes.

huh

And as I mentioned earlier, we are only now actually going to connect the process with data. If of course we could have done that before, could have done a zero measurement once, for example. Then of course you could have measured something and shown results after six months. That's just starting now, for my part anyway.

m

Yes, OK. Well, I'm actually through my questions. Is there anything else you would like to pass on to me? Or something you still want to share?

huh

No.

m

Okay.

Is there anything else I should have asked you about implementing improvements or things that could affect that process?

huh

No, I think most of it has already been asked.

Interview #5

m

Well, the first question: what role do you play in quality improvement in the hospital?

huh

Which role? I think especially if I identify something that I think oh, that could be better, that I then discuss it with the team or with the doctors and surgeons or with the nursing team. And then yes, also think about how we could improve that. And sometimes I leave that to the nurse. Because it's something they could pick up. And other times, that's something I can influence myself. And can improve yourself. And then I take steps in that myself.

m

Okay, so especially signaling things and passing them on to the people who can do something with them.

huh

Yes. And even if it is my area of ​​expertise, do something about it myself.

m

Yes. And I think you were also at those lunch meetings with the process analysis of the entire healthcare chain?

huh

last week, I did.

m

And what do you think of the process analysis?

huh

I want it myself. It was clear what the things were that were discussed last year. And whatever could be improved. And there were quite a few things that were indeed taken action. But they still need some attention. And of course some things are not so easy to solve. Which then takes longer. Yes, so yes, I thought it was clear in itself. Yes.

m

Yes, do you think it's something useful to keep doing?

huh

Well, I think so. If you really plan a conversation about that. Then everyone can also think about what I think could be done better in my area. And sometimes it's not things you always have control over. So if you discuss that with the rest of the group. Then there will be something to improve upon. Um, yes, I do think that it is quite surgically oriented. So the doctor's assistant from surgery is there. I'll be there, the doctor's assistant, the nurse specialist in oncology will be there. But I miss that sometimes it is from the whole chain, so to speak. So that the internist is also there and the MDL doctor. And yes, that sometimes it's a bit more... That it's a bit much surgically oriented. And so you also get improvements in that area. Which is of course good for me. But it's not over the entire chain yet, so to speak.

m

Yes. Is that something you would like to change about it?

huh

Well, I think so. Maybe then you can achieve even more policies to include their stuff as well.

m

Yes, exactly. Okay, and are there any other things about process analysis that you think could be done differently?

huh

Yes, I'm thinking about it for a while. I think for myself, so to speak. Is it good to talk to each other and then think about things? I think sometimes there are quite a lot of points. what we could do something about. And that you might ... say somewhere it is of course good to tackle all the points that are important. But on the other hand, sometimes it's a bit much. Which means that for some projects a little less is being done than we would hope. Because there are just too many things at once. Tell me where you could do what. And that it might be just too little invested in it, say.

m

Yes. Yes, okay. And if we look at decision-making, for example within the colorectal cancer care chain, but also in general when it comes to improvement projects. How do you think that is going at the moment? Will that be very structured? Are there meetings in which it is discussed whether decisions are made about improvement projects or does this take place on the shop floor?

huh

How's that going? I feel like things are being brought in at those luncheon meetings. And after that, eventually an Excel is sent. With the points that we are actually going to do something about. So um. Is that then?

m

You don't get too involved in that?

huh

No, I don't have that idea. In fact, I'm not much involved in that.

m

Those decisions are then made by the DB, I think? And you only get the result of that, so to speak.

huh

Yes. Yes. Yes that's right. Yes. I think that's how you go.

m

And um. If we look at the implementation of improvement projects. What are the steps normally taken? This of course differs per project. But broadly speaking, what are the steps that are often taken for implementation?

huh

Well it starts with that noting what is there. This is then introduced at those lunch meetings, I often run into this and that problem. And that a number of people brainstorm about how we can solve that and sometimes literature is also researched about how we could solve that. And then another proposal comes to the executive board. But I have to say that I actually... The only project I've been involved in is the obstruction protocol, which we evaluated.

m

yes I can remember that.

huh

Yes, exactly. And for the rest, uh. Now at the moment. uh. Not that bad

m

Is that done in a kind of working groups that people then brainstorm?

huh

Yes. We did that in a working group. Yes, with the dieticians and the physiotherapist and the nurse. And then evaluated how does that actually work? And is that satisfactory and what could be improved? So we did that in a working group. And that fed back to the day-to-day management.

m

And how was the communication afterwards in that project?

huh

I didn't really hear anything about it after that.

m

And that working group, that was really a meeting back then, wasn't it? Did that also go through the mail afterwards or were it just face-to-face meetings?

huh

Yes, we got together then. And then a few things done via email. And then finally there is a new folder and the protocol is adjusted. And that was how it had gone. Indeed.

m

Yes? Yes, okay, clear. And if we look at support from the hospital. Do you feel that the hospital supports quality improvement? And I mean through concrete means. So that there is time, money, personnel or certain training courses?

huh

Well, I do feel that since the colorectal care pathway. For example, that there is time to analyze things. And that the VBHC is also looking at this. And also if, for example, I want to have an order set adjusted for the colorectal surgery. That someone from EPIC has been assigned to us who immediately changes those things. Yes. Well, that's a lot smoother than it was before. Because you really had to create an order. And then each time you get someone else assigned from EPIC. And that has really improved. So I think they're supportive in that regard.

m

Okay

huh

For the rest, I think that the Executive Board notices this in particular. That support is given to them. At the things they want to do. Yes. So I notice that less.

m

And suppose you would like to implement an improvement project that requires money or whatever. Would you be able to get support for this from the day-to-day management or elsewhere in the hospital?

huh

I think I could just submit that to the daily management.

m

And if we look at the board of directors and senior management in the hospital. Do you feel that they are really involved in quality improvement?

huh

I think in the sense that they have made availability of these funds for the colorectal care path. That for example. Yes. For the rest, yes, I don't notice that immediately. But that doesn't mean they don't.

m

No, no, exactly. Clear. And how is quality improvement valued in the hospital. Is that something that people view positively or is it seen differently?

huh

Well, I think speaking for myself. I think it is viewed positively. Only that the Antonius is also a hospital. Which is often much improved. That people are actually very driven and want to improve many things. On the one hand, I think that's a great thing. Just say that people are willing to change. And be willing to do something somewhere. And on the other hand, that sometimes also ensures that there are a multitude of small projects. Those that just don't get off the ground well, say uhm. So that's the sad thing about it. But if I compare it to another hospital where I've worked, people here are very willing to change things. And people certainly want to contribute to that. Yes, sure. So in itself I think people are positive about quality improvement and quality projects. Only yes, maybe there could be a little more focus in these are really the main points that we want.

m

So the implementation could be a little better?

huh

Yes I think so. Yes.

m

Okay. You just indicated that people are willing to change if that improves quality?

huh

Yes.

m

Okay. And is that also something that comes from the DB. Or is it stimulated by the medical leader?

huh

Well, I think the board is very positive, for example about the obstruction protocol of course. And it's really progressing a lot. And that there are also other things that she really thinks could be improved, such as, well, the lab values ​​previously known in the department or further with the ERAS protocol. And she really conveys that to the team. uhm. So I think she does.

m

Okay. And if we look at the people who then sit together in such a working group or an improvement project. Are they often colleagues who already know each other? Or are they often people who have never met each other before?

huh

Well, so far I've done that anyway with a group of the surgical colleagues. So a physiotherapist I know. And also a dietician whom I know from our own, from the workplace. So those aren't different people than the ones I already know. No.

m

Yes. And the people you've worked with. Were they people with previous experience with improvement projects? Or was it something new to them?

huh

I think it was new to that group for some people. To handle it that way. Yes.

m

Okay. And how would you describe the collaboration? Is that going well?

huh

Yes, so I think that the collaboration with the people you see directly in the workplace, such as the dietician and the physiotherapist and the surgeon. That it actually works very well. Only that it is precisely with the people who are a bit further away. Like people who work in Leidsche Rijn. For example, the oncologist or internists or the oncology nurse specialist. That this collaboration is less of a whole than the people you work with directly. I also talk to the people I work with directly about things you think, oh that can be different next time

m

And if you look at the people who implement such an improvement project, well. Are the right skills available to implement that improvement in practice?

huh

Well, in some cases I think it still is, say that it is, for example in the group I functioned in that there are a lot of practically educated people and less university. As a result, it is less on a scientific level. And more on a practical level of what is useful for the department? And there I think that sometimes there could still be some support. For example, by supporting the academy by carrying out a PICO on a certain subject and in doing so. Because otherwise I would automatically have to be the one who does that in that group. While sometimes it is more a subject for dieticians, for example. To actually make a PICO about that. And that it is less within my field of expertise.

m

Okay clear. Are people compensated for the time and effort they put into an improvement project?

huh

Well, my experience is very often that, for example, the consultations also, now it was a lunch consultation, but very often the consultations of, for example, the colorectal care path are actually outside working hours. Yes, that makes for me, that is actually an objection for me to participate in it, because I participate in several care pathways, including pancreatic carcinoma. And also other improvement projects in the department. For myself I think it is important that it fits within my working hours and that it is not actually after five o'clock. My hours are compensated. But say in that sense. I get time for time. So if I work overtime, my hours are compensated. But actually I don't like doing that sort of thing outside my working hours. That makes it less fun for me.

m

Yes. But if there is something for such an improvement project outside working hours. Could that be compensated?

huh

I'll be sure to write those hours. Yes. Yes, OK. But then, just say I'll be there until seven o'clock in the evening, for example. While I would rather do that during the day. Those things.

m

Yes, exactly. Yes.

huh

So that costs you more in your private life than it costs you hours. But that will cost you something. Yes. Yes. Yes.

m

Okay. And is there a system with which you can collect data? Whether the data can be supplied for improvement projects?

huh

You can get everything from EPIC, but then you need someone... I've never had to do that.

m

You don't have much experience with that yet?

huh

No that is right.

m

And when I look at clinical care. Have the improvement projects. Did you really have an impact on the care that the patient experiences?

huh

Well, I think if you look at the obstruction protocol, sure. I think that's a real change. And I hope they introduce ERAS. That it is also that ... say there are certainly small things from ERAS that have already been implemented. Like spinal pain relief and things like that. They also all came from that colorectal care path, of course. That really has an influence on patient care and the length of stay certainly does.

m

Yes.

huh

So those are good things.

m

OK nice. One-to-last question. Were there certain factors that hinder an improvement project or make it easier to implement?

huh

uhm. Just think, yes there are always things that make it easier.... Things that make it easier is that the whole team is aware of a change that is about to be implemented. So that it is really supported team-wide. And that attention is also paid to informing all disciplines about a new protocol or working method. That helps a lot to get it all done.

m

So that there is support.

huh

Yes, there is a lot of support. And that it is also clear why you would like to have something changed. So that's for sure. For the rest... Yes that it is made easy for people to do something in a different way. So uh that you make sure that there are as few steps as possible in EPIC to arrange something. Or that it can be done in an easy way. And that every now and then someone reminds you of oh, do you mind that we do it that way now? Yes, I think those are helpful things. And that it is not just thrown over the bar somewhere. From okay from now on we'll do that and that. But that there is really also attention for its implementation and the memory of oh yes, we do it this way now. Because we think that's better there and that's why. Yes, so that's something that helps. And what doesn't help? Yes, actually the opposite. So if a plan is made somewhere and it's said that's what we're going to do. And the fact that it hasn't been discussed with the team is something I don't think helps.

m

Yes,

huh

And I also think what doesn't help is that there are too many projects at once.

m

Too much workload.

huh

Yes, too much workload and simply too little commitment. If you have to think about 1 thing, then you have more commitment to it than if you have to think about several things. Oh yes we do this now and we do it now, that might sometimes be a bit too much.

m

Yes. And then the last question is there one more thing you want to share with me. What do you think is important on this topic?

huh

Yes. Perhaps about the dismissal procedure. We had, of course, reintroduced this as a result of that survey. How we're going to do that now. And in that respect I did miss a bit. Also discussing how are we going to do it now? Because now it was actually more that they said yes, then the two of us now have to sit down at the bedside with the patient, ie ward doctor and nurse. So nurse together with a nurse specialist or physician assistant. And then we're going to do it like this from now on. So that's exactly what doesn't work. And so now it doesn't happen again. So I thought well, that's actually an example of nice work, say what you did to find out. What are the points that the patient has difficulty with? But not really, or at least too little, thought about the interpretation of how are we going to do it from now on? Yes, in the sense that it has been decided that we are going to do it this way. But that it is something that takes fifteen or twenty minutes per dismissal. And the question is whether it will really get that much better than what we are doing now. I find it difficult, for example, to give commitment to that. Because I feel like I'm already doing it in a pretty good way. Just not necessarily together with the nurse. So I think that's an example of this as well. Yes.

m

So there were still some objections, practical objections, and that should have been done more in consultation?

huh

Yes, I think so. Yes.

m

Yes. Okay. Well, thank you for the answers.

Interview #6

m

Well, the first question. What role do you play in the improvement team? I know there used to be in colon cancer there really was an improvement team. But now with the care chain there is a daily board.

k

Yes, I've been to those VBHC meetings. A number of times, also at those santeon-wide meetings in which the figures are then compared. I think those are the most important things.

m

And then a question about hospital support. What is the role of support staff is, say is, eh. Is the support and resources available from the hospital? To implement these improvements, so to speak.

k

Yes, I think so. So what. I'm not on top of that. But I feel that support is provided.

m

Yes. And also for you personally. If you sometimes have to do certain activities. In the context of that of the VBHC. Do you often do that during free time? Or has time been made available for that? Or other support available?

k

No, that's usually just all in free time. And last year I and someone together applied for the top care colorectal carcinoma. Yes. It was still quite a mess. we also did in our own time. But that uh. Yeah, you know, on the other hand, that wasn't something you could outsource either. Or uh. So that. Yes. It couldn't have been much different, I think.

m

Oh yes, no! Correct.

uhm. Okay. And then another aspect of the hospital. Do you have the feeling that the hospital board is involved in these kinds of improvement initiatives? And that also supports, so to speak. So really more from high up, the leadership of the hospital.

k

Yes, I think so. I think there is real attention for that and it is also hospital wide and yes. Even higher up, I'll just say Really a spearhead.

m

So, for example, do you have concrete examples that demonstrate this. Just say that there is involvement from the board.

k

Yes, for example, I have now started working at Poli directly. Once a week.

And that is of course what we actually do not do as freely established medical specialists,

because that is also in the region. And there is a non-competition clause. Was also from the board of directors and T and S. B. Was both the will to have someone at Poli directly.

To see if we can change things for colon cancer. So that way, yes it is supported. Then that was the question. From the NSB and the Board of Directors to

to go along with that. So that.

m

Yes, that's just another good example.

And then another question about the culture in the hospital.

Quality improvement and VBHC How is that valued in the hospital and in your own team? Is that a culture of acceptance and understanding or would you call it something else?

k

No, actually. Acceptance. And I have. I think everyone is open to that. And the people I work with are also motivated. Because that as a kind of tool that you can use. To improve care.

m

Okay. And is it something that is really appreciated by the staff. Or would you also say that people find it more of a burden. Then they have something to do with it.

k

I think they are always there. And for some people, it's just more extra work. But I think in general. Yes. Is it accepted that it is also something that can simply help you further and with which you can improve care?

m

Clear. And the next question. Which goes a bit further on this one.

The members of the improvement team and the other people involved. Have the desire and willingness to change things too. And also change their own working method, for example. As needed.

k

Yes, of course. And they also actively ask people if they can still do things. Or that they still have things to do with regard to the care of patients with colorectal carcinoma. So I think everyone is open to that.

m

And the medical leader, in this case our board member. Does it also facilitate and stimulate the improvement and change of, for example, working methods?

k

Yes.

m

Okay, then a question about the team members, about diversity and the like and experience in the team. Do the colleagues already know each other in the improvement team? If you look globally?

k

Yes, I think so. Yes.

m

Did they have previous experience with improvement projects?

k

Oh, I'm not sure. I didn't really have that myself.

m

And how would you describe the collaboration within the improvement team? With the different disciplines?

k

Yes. I think it is good. What I learned from the improvement team. Is that everyone is willing to contribute and also listen to each other. I think that's okay.

m

Yes. And for example if there are certain. If something needs to be sorted out. Or if you really need to put a lot of time and energy into something. Do you also have the feeling that the division of tasks is established. And that certain agreements that are made are actually carried out.

k

Yes, I think so. Recently we had an evening with a number of presentations. Then they were also asked to present it. By several others. And I think everyone then made the presentation. And contributed.

m

Yes. So it's not that just really sticks to meetings. But also really things are implemented and executed.

k

Yes

m

Okay. Clear.

And then the next question we actually talked a little bit about. Are improvement team members compensated for the time and effort they put into it? Are there financial incentives?

k

No, I do not think so. No.

m

And then as far as the data is concerned, there is support for supplying the necessary data. Systems or infrastructure available to collect the data?

k

Yes, I don't do much with that care path itself. I'm not very actively involved in that.

But also for inclusion in studies, for example. What we participate in. There is support for that. For example, the oncology nurse. So I think if you want something then it is best to get support for that.

m

Yes. So structurally in the form of an oncology nurse or, for example, research staff?

k

Yes.

m

Yes. Then another question about impact.

What impact, would you say, have interventions had on real clinical care in the hospital when you look at the colorectal cancer improvement cycle?

k

Yes, I think that will take a while. But I think mainly that obstruction protocol and also the prehabilitation. I think that in recent years has had the most impact on better surgery results

m

And you mean especially more postoperative outcomes or also impact on other things?

k

No, I think it's mainly for the postoperative outcome.

m

And if you also look more at, for example, patient satisfaction or patient experiences. Do you see a difference in that or not so much.

k

I think that is difficult for me. Because we are actually at the beginning of the process. It's bad news after the scan. And we see the patients again at the outpatient clinic when they have been discussed in the MDO. But then they disappear a bit from us. Yes and those two conversations, that is often not the same MDL doctor. So yeah, I actually just know more.

m

Yes. No, I get that. Because you are only part of the whole process.

k

Yes.

m

Okay, um. And then another question about unexpected effects. Were there any unwanted side effects from interventions that were implemented?

k

No, I do not think so.

m

And were there factors that facilitate or hinder the implementation of improvement initiatives in practice?

k

I don't think it's being thwarted. yes, I think, but that's true, I think for all specialists that time is the biggest problem. Yes, to find time to do things.

m

Yes, and actually more like the workload. That some people might want it. But just don't have the time to do something on the side to improve.

k

Yes. And also have many other tasks within the board of the benchmark or committee.

Well, you name it. Yes. Look, if you ask me personally. But I think that might be the case for many more people. I think that's actually the biggest struggle. And that doesn't necessarily have to do with the improved team. Or actually not at all. But that's more that every now and then you just have a lot of balls to keep in the air. And then? Yes, sometimes things go wrong when you would rather not.

m

Yes. So basically, then you have too much workload. Then there is not really, say, time made available to do certain things structurally. Like improvement projects?

k

No. And I don't really know if there's really anything there, shall I say, what I could do.,

No I have. I do the tasks I get. I do, I'll just say. I won't let it slip. But, yeah, I guess time eh. Time is an issue though.

m

Because of the involvement with the improved team. Would you say that your workload has increased as a result?

k

No, I do not think so. I think my involvement so far has been quite limited. So me, I often come to meetings, recently I gave a talk. We sometimes have meetings about things. Yes. But the home tax is very limited so far. That work is fine.

m

Yes. This question may be less appropriate. But let's see how far we get. What do you think of the way of decision-making within the improvement team? Why is a certain intervention chosen and not the other? Were you involved?

k

Yes, actually not. Not really.

m

And the last question about implementing an intervention.

How does that work? How did that come about? Was that an organic process or was there a structural approach to determine who does what?

k

a bit organic and also just okay, you know that there is a concrete question from do you want to do that? And one question I remember with that I. I also sat with her once. What was that called again? That the entire care process was mapped out. And the process?

m

Process analysis? Do you mean that?

k

Yes, that process analysis. With all those exits, shall I say. That's a big diagram, actually has the whole care in view so to speak. Yes. So that. We were then asked to do that. And no. So I think there is some direction from above. I think that's good too.

m

Yes, and that process analysis. What did you think of that?

k

Yeah, I don't really know that. I think that's good to get some sort of picture. And also if you look at that patient journey that we also talked about. That it can help. But I, I found it, seemed like a big job to do that. Yeah, I don't really know what it actually yields in the end.

m

okay so, because it is indeed something that is fairly new. That was not always done. And now we're looking to see if we can get useful information from that as well. Yes, if I understand correctly. Then you will find it interesting. But yeah. Do you think it will also take a lot of time and effort. And you're not sure if it really useful things come out, say.

k

No. If I only look at practice. Then of course that is the process that the patient ends up in. That actually goes super fast. Within a week and a half they will know the diagnosis and how things are going. And then, a short time later, they are operated on or not. So I don't think there's much more to streamline that. But yes, maybe if you combine it with patient experiences. Or with feedback from patients. I'll just say that that is such a process card. That it might help you.

Interview #7

m

Well, the conversation is now being recorded. Well, I just briefly told you what it's about.

First question. What role do you play in the improvement?

J

What is my role in the improvements?

m

Yes.

J

Yes. I think, among other things, consultation hours that we do for bowel patients. We see the oncological and non-oncological patients. We see the large ORs in advance at the consultation hour. We can certainly point out a number of things in that. For example, if people eat poorly, they are well fed beforehand.

J

And what we also do more often is socialize. So if things are playing at home that we can respond to. For example, if it is a young lady or one with a large family, for example, I ask how they have arranged it all for home. And then we need some guidance with that. So well, that way we're trying to see if we can tackle some things in advance. Which makes us. Yes, so that it is properly accompanied for during the recording.

m

Yes Yes. So, for example, with regard to new improvement initiatives. Within the healthcare chain and the VBHC?

J

we have also set up the care card. Again. I think that is beneficial for the healthcare chain. That we then discuss what the patient can expect after the operation. So the mobilization, the recovery of food and drink around the pain relief. So we have also re-introduced the care map of the care chain. Yes.

m

So if you understand correctly, you are more concerned with the implementation of various improvements within the healthcare chain.

J

Yes Yes Yes. Before that we suffered a while before, yes before the corona we started it with different disciplines together. So that includes the spirit care, the physio, the dietician and well, people from the doctors too. And department head. They were together from the care chain. And then we also had those brainstorming sessions. And a number of points have also emerged.

m

You mean the process analysis? With all those different steps. And that flow chart?

J

Yes. Well, we looked at that. That was then fully explained in six topics from my head. Yes. And yes, I don't remember exactly. Has faded a bit. Recall number of topics discussed at a time or one topic at a time

and in that of ok, what can we improve in that? So there were a few points. Yes, and to get started with that. So I think there is certainly added value in the entire healthcare chain to look at all of that. So that there is a better flow of a patient from the GP to discharge again.

m

Yes. Yes. That was actually my next question. What did you think about the process analysis? But if I hear it that way, you're positive about it.

J

Yes. Yes, of course. And I think that with that, that screening too, huh. What they created. For the older people who have a screening. That he is done beforehand from him. Shouldn't someone first strengthen themselves before he goes for OK. Or be in a good nutritional state. So no. screening is also a very important point. It has also come from my head for two years now. We've been doing that for two and a half years. No, that's also a very important point. As a result, people are no longer operated in the standard way. But that this is looking at hey, what does that gene have to gain from mobilization? And to make sure they go into the OR as fit as possible in a kind of quote.

m

Yes. No, exactly. So that. That certainly contributed to better outcomes. Yes. And then back to the process analysis. Do you think that in the current working method that is something that is feasible to do every so often, so to speak. Or don't think that often, so that something was a one-off.

J

No, this certainly is. But this can be expanded even further. I think there is even more value to be had. By also going to other hospitals to see what they do there and we can take things with us. So I think they are definitely not done with this yet. And I certainly think we can still achieve things. sure. Yes.

m

So you say it can be repeated, that process analysis and it can even extend to other hospitals. Yes.

J

Yes. To see what they do about it. Or are we way ahead of the rest or uh? Look like for example, I learned through another hospital. We don't do that here. Is that the patient walks to the OR from here. Or from one place and that's you, so they're put there on a bed that way. But then they walk to the OR. We don't do that here. But those are small things and are they feasible? Are those things for us? Or can we still make an improvement plan? Yes. sure.

m

Okay. And then the decision-making within the care chain or with the improvement team. Yeah, I don't know how much you're involved in that. With the decision to implement a new improvement, for example?

J

Well, I'm going to.. And yes, you know, that healthcare chain was a bit all on its ass because of the corona. It's through. Progress has been made, however. But I wasn't very active in that. I did do certain things. But that's also the activity you put in, I'll just say. So I will eventually get a certain decision. But I wasn't there at the time.

m

Yes, okay.

And in terms of implementation of improvement initiatives. You just said you're very busy with that?

J

Yes. Like the care card, I think it's a good thing that we've implemented it again. Yes. And I think that's also a positive point. Yes.

m

Yes. And what steps are taken to implement a particular intervention. How does that come about? Is that more organic or is that more of a structural process? That people are then designated from you will carry out this project.

J

Well, it's more of a structural process. I'm guessing from one of those meetings we've had before. That points come out of this, that the people who bring them in can then get to work with them. Have a look. What can we improve. So uh. At that point, people also feel responsible for doing that, huh?

m

Yes, OK. Well, some more questions about the hospital.

How has the hospital supported the project? Were there support staff? Were there resources, time, money or other things available to make improvements?

J

Well, I also think what's especially important is to see what the doctors are doing? How active are they because of themselves, yes, how do you say that to put themselves in the spotlight. And how active are they themselves with all the up-to-date stuff I'll just say. I think that's especially important because, uh, that's just how the care chain looks at what can be improved. So yes.

m

And did you have the feeling that the doctors did too. d

J

Yes. I certainly think so. And yes, there is then at that moment eh look certain things are good, I think to see what can we get out of it? And there is money available for that. But it is no longer about sitting back, but looking ahead. What is there to achieve? What are the latest figures? What can we improve? And that's where the hospital makes, does participate actively in it. Yes, definitely progressive.

m

And if you look at concrete resources. For example, is time really made available or is there a budget for certain things?

J

Yeah, you know, I find that a little tricky. Yes, you know, time is difficult if you have the time and can make time for it. There is certainly room to do that indeed. And in some way money is available. Sure, yes. If it shortens the length of stay, there is always something, in one way or another. Look if, for example, a care card is made. Then that can be done just fine. The hospital just pays for that. So sure. Yes.

m

And what about certain courses or training?

J

Yes. Well, you know, of course it is. There is room for that. Yes. Look, because of corona it's all on the hole. But there is certainly room for that sort of thing. And that money is also outsourced. Yes. Yes.

m

And more in terms of hospital senior management such as board of directors. Do you feel that they also support this, that they are also involved?

J

Yes, I don't know yet. Yes, you know, they do get involved from the doctors. Do they involve doctors from or the board of directors of is this and this possible and can we do this. And I think they are involved in that. But as a nurse I have little to do with that. So I think that's more of a .. that's more up to the doctors of hey. Can we do this and this And when the length of stay has improved, we can always talk about it.

m

Yes, exactly. That's right, And then another question about quality improvement.

How would you say that is valued hospital-wide and also in your own team?

Yeah, is that kind of supportive. Is there a culture that supports that?

J

Well, look. There is to see what can be improved I will say in the team. That is certainly being looked into. We have the expert teams in the hospital and also on the ward.

And that. That is certainly there to see if things can be improved. So we are actively working on this with the nurse. Yes.

m

Yes, but I mean more a little informal. Is there a culture, say in the hospital or in the department in which improvement is encouraged?

J

Secure. Yes, from the mt. And the department head. Yes.

m

Yes clearly. And when I look at the improvement team itself, so the different people who participate in it. Do the various members really have the desire and willingness to change themselves? And is the medical leader, does he also encourage that?

J

Oh, well, you know I'll fill it in for people that they will. I mean, people are always ambitious to adapt things, to change and I will say the MT and the department head are certainly prepared for that that there is room for that. Yes.

m

And the members of the improvement team did the colleagues already know each other?

J

Well, the members of our team do, those of the Expert Team. But on the others around the members of the care chain. Yes then. They know each other there. But it is not the case that some of them work very closely with each other continuously. It just depends on what your role is as a nurse.

m

Yes, exactly. Did the members also have previous experience with improvement projects?

J

Whether other colleagues have previous experience with other projects? Yes yes.

m

I know that such an improvement team is not something completely new.

J

Oh, no. There is always something that can be improved.

m

Yes. And if you look at the collaboration within the care chain or within the improvement team. How would you describe that?

J

Well, I think fine. I think uh the corona has made it all a bit different. And in a different way so to say. But I think that there is also just good communication with each other. Yes.

m

Okay.

And in terms of skills, do the people in the improvement team have the right skills to carry out interventions well?

J

Yes. I think that's fine yes.

m

And that's what we just talked about a little bit. uh. Are team members compensated for the time and effort they put in and are there financial incentives? You already indicated that there is a budget for courses and to print certain posters. That there is a budget for that.

J

Yes, be sure. It is also considered if you are involved in a project group all day. That there is room for that. That you can sit on it. Yes. And the one that it is depends on the grid. And how the staffing is. But there is certainly something to talk about and negotiate about. Yes. There is room in that. Yes,

m

but no other financial compensation or financial incentives?

J

Yes it is not that you get an extra bonus. No, it's just part of your job.

Otherwise you don't have to work in healthcare.

m

Yes. No, you won't get very rich from eh.

J

No, it won't make you rich.

m

And then the data infrastructure

Is there a system available with which you can collect data?

J

al. There is, I think. But I know I won't work with it any further. But there is, you know. Yes it is. That's for sure. Nursing specialists do much more with that.

m

Okay and in terms of impact. Would you say the interventions that have been implemented so far. That they have really had an impact on clinical care?

J

Well, I think some things have improved. Indeed. And I think it may have shortened the VOD. Yes. And that a patient will go into surgery stronger. Yes.

m

Yes. Okay, yes. And were there any unexpected effects of such an improvement? Unwanted side effects?

J

Well not that I know so. No,

m

Okay. And then if we look at factors that, for example, facilitate or counteract such an intervention. Are there any?

J

not that I can name that.

m

Okay. And if we look at the workload, it increases because you are working on improvement projects.

J

Well, in the end I think the processes are getting better, indeed. So there will definitely be some room for it. I'll just say it improved. So we will definitely notice people.

m

Yes, but I mean more about you personally. That is because you are now also working on the healthcare chain and those improvements. Do you have the feeling that your workload has actually increased?

J

The workload has increased.. or what did you say?

m

The workload

J

Or has that increased? Yes. Well, look, um. I think it is the period in which you are around the patients. When we just started, the second wave, the pressure on the beds is very high. And also on the surgery beds. So yeah, it's a bit tricky to compare. Whether that really is the workload, or it or the things you enter actually reduce the workload, I do. uh. Yes, I don't know. I think people. I think it will eventually improve because people are on their feet faster. Because they know they have to mobilize. That uh. Do you know. There are more, I think because they are better informed. If there is, I would say that there is an improvement in patients because people know more. So yes, I think there are improvements. Yes.

m

So you might have a little more workload in the beginning to implement such an improvement. But then it will eventually make the work easier and you will have less workload.

J

Yes. Yes. Well. And that people also know that they, look in the past, they were all in bed for a long time. And nowadays people have to get out of bed faster. And again they can, they have to be able to eat and things like that. So yes, that is. It does change I would say.

m

Okay clear. I have all asked questions. Do you have any other things you would like to share with me?

J

No, I think it is good that such a care chain exists. And that you can see with different disciplines that it is being improved. So I'm definitely in favor of that to see if some processes can simply be better. And I think you get more value out of it in the end. Through a better flow of all kinds of things. Sure, yes.

m

Yes. That's why that's very good, we've been working on this for quite a few years now.

J

Yes. Yes. And I had just hooked up. But also because of that Corona has also been a bit on his ass. Some things. No, I have no other things.

m

Okay. Well, thank you for the time.

Interview #8

m

What is your role in the improvement team or in improving healthcare in general in the context of Value Based Healthcare?

R

Which role? Yes, I am also on the care chain board. And I am the replacement for the chairman, Anke Smits. And I think that's the most important role.

m

Yes. And are you really working on certain improvement projects yourself? With implementing?

R

uhm. Yeah, I'm generally busy with… I'm a bit lost what that's called. With the general practitioners who would still like to be aware of many things in the healthcare chain and to think along and participate in this. And.. I don't remember what that project is like, but I'm still working on that. We have discussed it with the general practitioners and the patients.

m

Yes. And are you also involved in the value-based healthcare process from Santeon?

R

um. Yes....

m

With all those different hospitals that come together. Do you go to those meetings?

R

No, I haven't actually been there.

m

Okay

R

But in the context of ERAS 2.0, I actually want that. We have agreed that I will go to such a meeting and the Martini hospital.

m

Yes, okay. Um clear. And have you been to those lunch meetings from a while ago? The entire healthcare chain and all those different steps were mapped out. And that a real process analysis was carried out. Have you been to those meetings?

R

On Tuesday?

m

No, that was a few months ago. That a kind of Flowchart had been made for every step in the care chain. And then people were invited and we asked at every step of what are any improvements? Were you there?

R

Yes Yes Yes.

m

And what did you think of it? Did you find it useful to do?

R

Yes, that's super helpful. Very helpful.

m

Yes. And do you think that's something we should repeat regularly. To continuously improve care?

R

Yes I think so. You have to evaluate that anyway by… If you repeat it, you can evaluate it too. It would improve a lot.

03:42

Speaker 1

Yes. And that process. Other songs. Is there anything you would like to change about it? Or do you say it's actually good how we're doing it now?

m

Yes, and that process analysis, would you like to change anything about that? Or say the way we do it now is fine.

R

Yes… I'm not a process analyst. But I think the way we're doing it now is fine.

m

Okay. Because yes, some questions are indeed more or less applicable to you. But these are the standard questions I ask everyone. Then we move on to the next question, decision making. How is the decision-making process going at the moment? When it comes to implementing certain improvement projects. Or are things changing in healthcare, are you involved? Are there specific meetings for that, for example?

R

uhm. Well, look, we have... that that goes... Yes. We do this in the group with the colorectal group. If we have an idea to change things, it is always discussed in our colorectal consultation. Because once in a while we have. And now, but nowadays with the care chain, that is always included in that care chain. Yes, I am working on a project myself, in which we have noticed that the MDL has much less supply of colorectal patients. And if yes, then you set up a working group for that. And then you can also use the people from the care chain. And people from lean and stuff. So, uh, that's helpful.

m

Yes. Okay. So in itself that is just transparent and decisions are taken together in a group.

R

Yes Yes.

m

Okay clear. And when it comes to certain improvement projects. What kind of activities are undertaken to actually implement a particular intervention?

R

uhm. Yeah, you mean practically how that goes?

m

Yes, more generally. Because that also differs from project to project, of course. But in general, are there certain steps that are often taken to actually implement something?

R

uhm. Yes, I'm thinking about which projects… For example, the project, I think that is an important project. That he and I are going to operate on a robot. That is of course an important step. This is first discussed in the large GE group. And then discussed in the colorectal subgroups. And then it is implemented in such a way that we can get started with it.

m

Yes. Okay, um. And that means that you are going to operate with the robot?

R

Yes. Yes.

m

That's a very specific example. For example, are there other projects where things are being changed in healthcare? Is that picked up organically by someone? Or is that a division of labor? How am I supposed to see that?

R

Yes, I think there is a division of labor. We have... You're not always at the colorectal meetings. But then at least it is said that now you can do that .. Everyone then indicates what they want to find out in order to possibly change and we give everyone a task for that, yes.

m

Yes. Okay. How do you view support from the hospital when it comes to improvement projects? Is there support in the form of staff or resources to do things?

R

Yes. That has improved a lot with the healthcare chain, hasn't it? Because uh you can really ask the people from the healthcare chain to help change things. Yes, so that's it. I think that's good support.

m

Okay. And if you look at the hospital board. Are they also involved in improvement projects? Do they also invest in it?

R

I think they are definitely involved. Uh Invest. Well, look. If you're talking about the robot, that's an expensive treatment. So they are definitely investing in that.

m

Yes. Okay. And how is quality improvement or value-based healthcare valued in the hospital and in your own team? Is that something that people find important or valuable? Or do people see it differently?

R

Yes, that is a widespread concept throughout society.

m

So is it appreciated?

R

Yes, of course.

m

Yes. Okay. And is there also in the partnership or in your own team, the desire and willingness to change. To do things differently. If that improves the quality?

R

Yes sure, sure.

m

Okay. And, for example, do they facilitate and stimulate this from the care chain for dam cancer and from the day-to-day management?

R

This is done somewhat less by the partnership, but it is certainly done by the care chain.

m

Do the colleagues who are working on improvement projects have previous experience with this? Or is this actually new to them?

R

I don't think most people have that much experience with that.

m

No, but is this something completely new for them?

R

Well, I do believe in some departments.

m

And how would you describe the collaboration? For example, if you want to implement an improvement project. For example, you work with another department or with a different discipline, is that going smoothly or..?

R

Yes. Yes, everyone who thinks it is very worthwhile to put energy into that. So that works well.

m

So there is good cooperation in the hospital.

R

Yes sure, OK.

m

And it is often colleagues who already know each other. Or do new people come together to implement an improvement project.

R

Yes, you often know each other. But sometimes you also have things you do with the lab. And yes, those are usually the people you don't really know that well. So, uh, sometimes those are strange to you, yes.

m

Yes. And if you want to implement an improvement project. Are the right skills available to carry out the intervention?

R

uhm. Yes I think so. It doesn't always go as fast as you want. But I do think those skills are there.

m

Yes. And are there financial incentives? Or are people compensated for the time and effort they put into an improvement project?

R

No.

m

So that actually happens on your own time.

R

Yes,

m

Okay. And speaking of the dates. Is there a system available with which you can collect data or use it for an improvement project?

R

uhm. Well, there is a department that you can ask for data.

m

Which department do you mean? The IT?

R

Yes, the IT. But also that lady who can look up and interpret all that data.

m

The data analyst?

R

Yes, yes, yes, indeed. That's very helpful. Yes.

m

Okay. And if we look at the impact on clinical concerns. uhm. Do you feel that the improvements that have been made have really changed clinical care? Or really, Have improved?

R

Yes, of course. Secure. Yes.

m

And in what way? Do you have an example of how that has had an impact?

R

uhm. For example, the ERAS. That's people all this going home much earlier. The robot once again indicates that people are hospitalized for less time. Yes. Determining a CRP is faster, so that people are hospitalized for less time.

m

Okay. Most of all, patients notice that they can go home a little faster.

R

Yes. But also have fewer complications. We also had a project that we no longer give epidural catheters. That is a very big advantage for the patients. Because they always had low blood pressures, numbness in the legs and luckily we don't have that anymore.

m

Yes. That is indeed a good example of how care really improves in the workplace. And did some improvement projects also have unexpected effects that occur because you change something in practice?

R

I'll have to think about that for a while, but I don't think so.

m

For example, that you enter something and that, for example, as a side effect, the workload increases for the staff.

R

Oh, so. uhm. hmm. Well, okay. Yes, it would. That could be. There have been improvement projects in which, for example, stoma care was better taught by patients. So they can go home faster. And of course that takes a little more time for stoma care.

m

Yes, OK. And are there certain factors that facilitate the implementation of an improvement project? Or counteract?

R

Factors?

m

Yes, I'm talking about certain things that make it more difficult, for example, such as a lack of time or money or whatever. Or certain factors that make it easier to implement an improvement.

R

Yes of course. There is always a lack of time and money.

m

Yes, I think every doctor says that.

R

Yes, exactly

m

And other things that make it more difficult to implement an improvement, regardless of time or money?

R

Well, I don't think the persons, because the persons are all very willing to work with.

m

yes ok

R

Yes. I have such a project with the MDL doctors. I would like people to be seen faster by the MDL doctors. That the GP also refers to us more quickly. And there has to be a special computer program for that and there is no money for that. And there is no project manager for that. Yes, it just stays there. That is very frustrating.

m

Yes. That is indeed an example of having a good idea. But unfortunately you can't enter that in practice. And are there also things of which you say well, that helps a lot to be able to introduce an improvement?

R

hmm. Yes. I think the healthcare chain is very helpful, yes.

m

Okay I'm through with my questions. Is there anything else you would like to pass on to me that you think is important?

R

No, I don't really have any additions.

Interview #9

m

Well, the first question. What role do you play in the improvement team or in the colorectal cancer care chain?

W

What role do I play? I find that a difficult one, I don't have an official role, I'm not on the day-to-day management or anything like that.

m

No, I mean more of you are involved with improvement projects etcetera. So basically everything you do officially and unofficially to improve healthcare.

W

Yes. I think I am partly signaling, say where bottlenecks are. And try to link that back to the care chain. Yes, I have tried through lunch meetings, say that too, to reflect that. This has also been an important function of the lunch meetings, in order to identify bottlenecks. And I do indeed try to tackle and improve things by means of yes, sometimes small work groups or other things or just privately eh personally.

m

Yes, okay. Clear.

W

So especially signaling and trying to pick things up.

m

Yes, okay. and those lunch meetings and that process analysis, you were just talking about. What did you think of that?

W

I really liked those lunch meetings. And also nice to meet other disciplines, so to speak. And sometimes you hear yes… you were able to solve bottlenecks there very quickly. That's really nice, I think that's fine. You can also, yes, it's also just good to see who's all further down the chain. So I especially liked getting to know each other. It was nice that that was physically possible, at least for a large part as far as I'm concerned.

m

Yes,

W

You can actually lose your points there. And things can be picked up. I found those positive meetings.

m

Yes. Do you find it useful to add to the current working method, those lunch meetings and the process analysis?

W

Yes. I liked those lunch meetings. Certainly very useful. Yes. Yes.

And that is of course something especially in the beginning to start up such a care chain. But I do think it's easy to do that more often. In this way, of course, we also find out where we are now?

m

Yes, she would like to repeat it.

W

Yes. I do think it's good to repeat that once in a while, and then I'm not sure how much time, but once a year so to speak, repeating it. And then indeed looking back at well what have we achieved last year? And where are we now? Yes, and what are the points for the coming years. And it's good to do that in a limited group, I think. The difficult thing is that you will of course need many more lunch meetings.

m

Yes, because we had something like 5 or 6 lunch meetings in total.

W

Yes. But then you can focus more specifically on a number of aspects. Yes, so I think that's positive. The trouble is that you may have to connect four or five times yourself. Well, that's no different. Yes, I think I also have to be critical of what I do want to join and where I don't.

m

Yes, and would you like to change something about it? Or do you like it the way it is done now? The process analysis.

W

Yes, I always really like making concrete agreements with each other, eh. So I think it could be a little more specific in one way or another. So whether in the preparation something more concrete or uhm .. So that it is less noncommittal so to speak. Yes, so that really concrete goals are set, if that is possible. And also uh in terms of workgroups and things like that. That it is actually laid down even more concretely.

m

Yes, clearly. The next question: what activities are undertaken to implement an intervention or improvement projects in practice? And of course that differs from project to project. But more generally, what are often the steps that are then taken to actually implement something?

W

uhm. Yes, important of course to first inform the people involved about a new implementation. And assuming that there is already a new intervention or a new implementation.

m

Yes.

W

So yes. Inform the people involved. Make sure yes especially for clear instructions or materials. Or whatever is needed. A good evaluation. And, above all, feedback from the evaluations. Also think very important that the people involved also see what they are doing something for.

m

Yes

W

So if possible also give measurable results in feedback of what their implementation has done.

m

And those steps you just mentioned. How do they come about in practice? Who does that? Is that organic or is there some sort of division of labor?

W

Now in the practice of the healthcare chain, so to speak?

m

Yes

W

Yes, I think that a lot of things like that are thrown in by the members of the daily steering wheel, so to speak. And sometimes it is delegated, say to a working group.

m

Yes.

W

And sometimes it stays with the day-to-day management. That they take more responsibility for the implementation themselves. Yes.

m

Okay. That is often the day-to-day management. And sometimes also other people who then perform it on behalf of the daily management, so to speak.

W

Yes, in a kind of workgroup indeed. Yes.

m

And during an improvement project, how are all involved professionals informed during the project?

W

I think that will be very different per project. I've been thinking a bit about our residue constrained or obstruction protocol project. Then of course we have contact with each other by e-mail, so who wants to participate in this? And who has which role, you make an appointment to sit together. And yes, an appointment or a product will come out of that.

m

Yes. Okay clear.

W

I think those lines are pretty informal.

m

Yes, often informally. And often in the form of an email or meetings.

W

Yes.

m

Yes. Okay. And how does the hospital support improvement projects?

W

That's a very general question, of course.

m

To make it more concrete: so support from the hospital, I'm talking more about support staff. Are resources available? Time, money or training with which you can tackle things in practice.

W

Yes. Well, I think if I had a concrete plan. And I would knock on the door of the healthcare chain with that. That I can certainly get support from that. For example in the form of data via the managers. Also financial resources if necessary. uhm. Yeah, I don't have a concrete example of that right now. But I do have the idea that that is certainly available and possible

m

Yes, so there is support from the hospital.

W

Yes I think so.

m

And when I look at senior management and the hospital board. Are they also involved in improvement projects? Or at VBHC?

W

Yes I. Well, I have the feeling that they at least have a warm heart. To what extent is that direct involvement? I find that very difficult to assess. Yes, I don't know that well. I haven't had much to do with that myself.

m

No, okay, clear. And how is quality improvement valued in the hospital and in your own team? How do people view it?

W

Um, yes, of course everyone wants quality improvement. That is obvious. It is true that sometimes you get through the issues of the day. And due to the hustle and bustle of the day, we don't really think about it enough. And that you just keep going. And that there might be some snow under it because of that. But I think everyone is aware of the fact. That quality of care is important. And that you also want to improve that. But sometimes it is also difficult to recognize where there are opportunities for improvement.

m

So the practical implementation of quality improvement. The idea is therefore considered important, but implementation is sometimes difficult in practice.

W

Well, I think that sometimes it gets snowed under by the hustle and bustle of the day. Just say that you will continue in the way of working that you are doing. Yes and that sometimes it is quite difficult to change things.

m

Yes, OK. And is there a willingness and desire among staff to change? What if that leads to better care?

W

Yes, in general I think yes. Look, there are always people who lead the way with that sort of thing and people who have a bit of a hiccup with it. But in general I think people are definitely willing to make changes. If you also know what you're doing it for. I think that's very important. The purpose of a change must be clear.

m

Yes.

W

It's mostly, yes, I think so. Change must be concrete, be clear. And the goal should be clear. Yes, people are definitely working on that. Yes.

m

Okay. And the medical leader of the care path and the day-to-day management, do they also facilitate and stimulate this? So the willingness within the staff to really change?

W

Yes, I think they certainly encourage and facilitate that. What I sometimes find difficult is that uhm. I understand that such a day-to-day management is actually more for overarching ideas, say the vision. And that it is sometimes difficult who is responsible for the implementation or the next step. Yes. And maybe sometimes some lines need to be drawn a little more clearly. And feedback is needed to one of the daily precise members for example. And that you have to ensure that one of the people of the daily management is also responsible for the working groups or the initiatives that fall under them. Yes, that's clear too. Yes you should get clear feedback or whatever you want to call it.

m

Could that be even better?

W

Yes I think so. That's just my personal opinion. I don't have much experience with that either. Yes, but I have a feeling that maybe that… Hey, the enthusiasm is clear. The will to change that is certainly clear. But sometimes there might be something more concrete... I think I often call it concrete, but that's clear. That more concrete agreements follow from this.

m

Yes, that's obvious. And if we look at the people involved in improvement projects in general, are they often colleagues who already know each other or are they strangers to each other?

W

Yes, I think you often do that with a club that you already know and I think that might be one of the tasks of the day-to-day management. To bring together people who don't know each other that well yet. That's also the nice thing about those lunch meetings, of course. Anyway, you have to put in a little more effort to work with strangers. So I think that the day-to-day management could perhaps play a little more role in that. To bring that together. Yes.

m

Yes. And those people who are involved in those improvement projects. Did they have previous experience with quality improvement or carrying out improvement projects? Or was it something they did for the first time, so to speak.

W

Yes, I think that's a bit variable. Certain people are more likely to get involved in these kinds of things than others. So there will often be people... for example, when I look at the department, they are often the same people, of course. Yes, but I think that will vary.

m

And how would you describe the collaboration while implementing such an improvement?

W

Yes, that also varies per project. But I do think that in that sense the healthcare chain ensures that you are all on the same page, so to speak. That is very widely disseminated. And I think that as a team you also feel that a bit more, so to speak. That you all want to go in the same direction. I think that is also the added value of such a care chain.

m

So yes, as a sort of summary of your answer I would say the collaboration differs from project to project. From good to less good.

W

Yes, but I do think that the fact that it falls under a care chain. The fact that this does mean that as a working group you have a bit more of a feeling that we are going in one direction together.

m

Yes, I understand. Okay. And if we look at the skills to implement an improvement project in practice. Are those skills present in such an improvement team?

W

Yes, I think that would be very variable too. And that of course depends on your improvement and..

m

Yes, it is also a very general question.

W

Yes Yes Yes. I think it is sometimes difficult to get enough spin. Yes, to find sufficient support in the end for an improvement.

m

So that's an important skill. Creating support.

W

Precisely. Yes Yes,

m

Yes. And is that something that is often missing? Or is that something that is going well?

W

Well, maybe that is. I always find that a critical point. Yes, to have sufficient support. And that sometimes it remains something that you come up with as a group. How do you make sure that it... Yes, that everyone can work with that too, see the meaning of it.

m

Okay. And are the team members compensated for the time and effort they put into it, for example are there financial incentives?

W

Not as far as I know.

m

Okay. And there is also a system available with which you can collect the data you need for an improvement project. Or who can provide the data you need?

W

Yes, I think it certainly can. In this way we can request data. And I think if you don't know that road yourself. That this can also be done through the day-to-day management.

m

Okay, so there is a good data infrastructure.

W

Yes I think so. Not all data is of course good, or readily available. But that will also vary by subject. If data is available, it can be easily obtained through the healthcare chain, so to speak.

m

Yes, okay. And the interventions are those that have been implemented in the past. Have they really had an impact on clinical care?

W

I find that a difficult one. I'm not really sure which interventions were ultimately implemented in the healthcare chain. Perhaps more so at the nursing ward level. So I don't really know enough about that.

m

Okay. And were there any unexpected effects of implementing an improvement in practice, as far as you have experienced that?

W

Well, I don't know.

m

No. Okay. And if you want to enter an improvement project. Are there certain factors that counteract or facilitate this?

W

Yes. Then you have that support again. I mainly think what is important.

m

Yes. So that can either facilitate or hinder, depending on whether it's there or not?

W

Yes, I think so. And the same also applies to a bit of work pressure. At the moment when everyone, especially with corona, has had a bit of a crazy time in that regard. And being very busy with other things, then you are not so open to changes. And then that will also be more difficult to implement.

m

Yes, so those are barriers for an improvement project.

W

Yes.

m

And are there factors of which you say, well that helps a lot to implement an improvement?

W

Well, I think especially if you expect a big effect. Yes, so and if the workload is lower. Yes, that is of course always pleasant for everyone, but also if a clear effect is to be expected.

m

Those were actually my questions. Then I have one last question: is there anything else I should have asked you about this subject?

W

No, I find it quite difficult. Because of course I'm only indirectly involved. Yes, I am involved with the colon cancer patients. But in the care chain and in all projects, yes, very indirectly. So it always remains natural from a very limited side that you answer.

m

No, but that is why I also do these interviews with various people, specialists, nurses, nurse specialists and other people in the healthcare chain. To get as many different perspectives as possible so that we get a total picture.

W

Yes Yes. No. Because I do think that the care chain does have a positive and stimulating effect. And I think her enthusiasm is certainly very important to mention. Yes. And on the other hand, that also immediately ... is that also a pitfall so to speak. That by being very enthusiastic you want to tackle many things at the same time. And that sometimes you also have to have a good prioritization. Well, I want to tackle this first. And if that works, we'll deal with it. Yes. And that you therefore need good feedback from the various improvement projects.

m

Okay. Then I want to thank you for the time and for your answers.

Interview #10

m

The first question. What role do you play in the improvement team, specifically for colorectal cancer?

N

Okay. Well, yes, I am involved in value-based healthcare, so involved from the healthcare chain. And I have been busy with the care path colorectal carcinoma. That in itself is an improvement I think. So those are 2 things I did.

m

The first thing you said is that you are connected to the VBHC colon cancer. So what exactly do you do in that?

N

Yes. We had a discussion from the MDL, in particular the diagnostics and the work up of patients. Can we investigate something there? And the early carcinoma.

m

Yes,

N

Those are the 2 most important pillars for my field.

m

Yes. Okay clear. And I think you were also at those lunch meetings then, where the process analysis was done. So that all those different steps were mapped out. What did you think of that process analysis? Did you find it useful to do?

N

Yes, I really liked it. Yes Nice.

m

Yes. And is that something we should say structurally? Or something we should repeat?

N

Well, actually we have yet to get feedback from that. If we know what the results of that are. In my own field, but also for others. If you don't hear anything back, it's not that useful to do a new session.

m

Yes, so you mainly want to hear what comes out.

N

Yes, so you want to have information exchange and feedback, then you keep people motivated. I actually never heard back from it.

m

Yes, exactly. No. Okay. So that's something you might want to change about the method. That there will be more feedback to the people involved.

N

Yes.

m

Okay.

N

Yes, that is already happening a bit, we get the newsletter. But, yes, that's it.

m

Okay. The next question is about decision making. What do you think of the decision-making process in the hospital at the moment? If yes, improvement processes or improvement projects? Is that going well? Do you think that could be better?

N

uhm? Yes. Do you have concrete examples of that? I find that very complicated. Are there things you mean?

m

Yes, more generally, if, for example, an improvement is selected to implement in daily practice. Is that something that happens organically, or is it a structural process?

N

Well, yes that changes sometimes. Sometimes little things go well. I notice that if we want to change big things, it takes quite a lot of energy from those involved. So I think it's always a trade-off of how much we want it. And how much energy and time are you willing to put into it? On the one hand, we have introduced that care path. The people who are committed to this cost a lot of time and energy. But we still wanted it to come.

m

Yes.

N

But if you look at how things are going, there is still a lot that can be improved. For example, there is far too little use of the dashboard. We get far too little of what's in it, so to speak.

m

Yes.

N

I have the idea that that often ends up on the specialist's plate to crank it up again. And that is also difficult then you think, yes I have to put time and energy into it again.

m

yes, but now I think you are also talking more about implementation of certain things.

N

Yes.

m

But that process before that, so before you start implementing something, but more the process of the decision-making itself. For example, from OK we are going to introduce a new care path, or we are going to introduce something else. How do you think that process is used to make such a decision?

N

Yes, that is not always transparent, so to speak. I don't know where those kinds of decisions are made.

m

Yes. Okay. So you are not very well informed about how that goes. That's a bit of a black box, so to speak.

N

Yes.

m

Okay clear.

N

And I'm not involved in everything either. So I don't need to know everything about everything.

m

No, exactly.

N

If new agreements are made about the AGE MDO or new agreements about the operated patients on the ward or the anaesthetic, we do not need to be involved.

m

But would you like to be more involved in decision-making? Or do you think it's good that you don't have to be everywhere?

N

Well, it is good to get more information and more insight into what is happening and what people are doing. It's nice to be involved in that.

m

Yes. And then let's talk about the implementation. You just said that it is difficult with large projects. And that often the specialists have to crank it up anyway.

N

Yes.

m

And if you look at how such an implementation works, how does it come about? Is there a good division of tasks, or is that also a bit organic, that people pick things up themselves?

N

Yeah, it's hard to talk about something so abstract.

m

Yes, I understand. It is indeed abstract. That's why I'm trying to gain more insight into it.

N

We are now working on the regional MDO of colorectal carcinoma. And that's pretty transparent of what's happening and why things are happening. So that in itself is reasonable.

m

Okay, um. And if we look at the support from the hospital. Do you feel that the hospital really supports improvement projects? And really make resources available for that?

N

Yes, many resources have been made available for VBHC Santeon. From my perspective, as an MDL doctor, I don't think it makes much sense. I think that if you put all the money and all the people involved in that VBHC project, if you put that in our care path. Real digitization and ease of use, etc. that that would be better spent. Then we don't need VBHC, because then we can read everything live from the dashboard.

m

Yes. So you're actually saying that you would like to see more in daily practice, say, that the money is used to do concrete things. Like setting up a care path.

N

Yes. I think the hospital would like to do things with quality and improvements. But they are guided by things they are used to from different organizations screaming loudly about what needs to be done. But if I look at reality, what does the patient really benefit from? It really benefits the patient if the care path simply generates an MDO report very tightly, which continuously keeps the GP informed of what is happening. If they continuously know what the waiting times are, and are continuously informed about complications, that really benefits the patient. And that VBHC session in which half an army of ICT has been working to get data from 2016. The patient has much less of that.

m

Yes, clearly. I know what you mean. And in terms of support staff, is that also available for improvement projects? Or do you have to do everything yourself?

N

Well, I have to say that the care pathway was quite well supported.

m

So there is staff available who can do things.

N

Yes. There were staff for that. Yes, but when it was done it was completely released. There's no one there to monitor the care pathway, right? And ask people how is the user-friendliness and what can we improve. That new initiative and the maintenance of such a care path yes, that is entirely up to the user and the specialist.

m

Yes.

N

And there… Look, if I want something, I can just call people again. And then I have to explain.

m

Yes. Yes.

N

If you, as a healthcare chain or as a hospital, want those care paths to run smoothly, see that people also reap the benefits. Yes, you have to put someone on that.

m

Yes. So structurally someone who monitors this.

N

Yes. And that care path, I dare to say that, it works. But I think patients could have benefited much more from it.

m

Yes. Okay. Clear

N

And that's where the hospital's commitment is really lacking.

m

Okay. And if we look at senior management, ie the Board of Directors, are they also involved in improvement projects?

N

Yes. Yes. Yes, of course.

m

Okay. And hospital-wide, but also just in your own team, how do people view quality improvement? Is that something that is considered important? Or what is valued?

N

Yes, that is considered important, it is appreciated. Yes, of course. But apart from that, there are an incredible number of organizations that consider their own quality project to be the most important. There are so many agencies that scream about what needs to be improved in the hospital. So there is some registration fatigue.

m

Yes.

N

So new projects are always welcomed with a sigh. Because previous projects, read VBHC, read care path, they are all not yet finished. So I think there is fatigue.

m

So there is some fatigue when it comes to new improvement projects.

N

Enormous.

m

Yes. This is mainly due to the fact that the previous projects have not yet been completed properly.

N

That we have not yet been able to reap the benefits.

m

Yes. Yes, exactly. You would really like to see more concrete results.

N

Yes, not for me, but for the patient.

m

Yes, exactly. Okay. If you also look at people's desire and willingness to change their daily work to achieve better results. Is that present?

N

Yes. Yes, of course.

m

So people are willing to change things. If that really leads to better results. Okay. And if we look at those improvement projects, more generally, are they colleagues who already know each other? Who then start up such an improvement project together? Or are they often strangers to each other?

N

Yes, I don't know. I think most people know each other, I think.

m

ok, so is there any previous experience with improvement projects? Or is this often something new for someone who is working on such an improvement project?

N

Yes, hard to answer. Nothing comes to mind right now.

m

Okay. No, that is. You may not be able to answer all questions. But that in itself does not matter. How would you describe the collaboration between colleagues when they take up a new improvement project. Is the collaboration going well or could it be better?

N

Yes, it's good.

m

Okay. So also between different disciplines or departments, is the collaboration going well?

N

Yes.

m

Okay. Yes. And if you look at the right skills to carry out an improvement project. Are those skills and those skills also present in the staff to properly carry out and implement an improvement project?

N

Yes, a lot of beach at EPIC huh. So yes you know there are very few people who have those skills. That is a bit general though, I don't know if this also applies to colorectal.

m

Yes exactly, some things are indeed more hospital-wide and not necessarily just for colon cancer.

N

Within the department there are a lot of projects that we want that really offer improvement. But that ends up with the implementation in EPIC. Someone has to fix that, someone has to fix that.

m

Yes. Exactly, you need specific skills.

N

So we run into that.

m

Okay clear. And if we look at compensation. Are people compensated for the time and effort they put into improvement projects? Are there financial incentives, for example?

N

There is no compensation anyway. And the financial incentive… no.

m

No. Okay, so basically you just do it in your own free time.

N

Yes.

m

Okay. And if we look at the data infrastructure. Is there a system available? For example, with which you can collect data that you need and supply it for improvement projects?

N

Yes, the structure is there in the form of the care path. But little or no use is made of it.

m

Yes. So that's more on the users side. That it is simply not filled in, say.

N

No, that's actually quite reasonable, almost with every patient. But it is not taken out.

m

Oh, it won't be taken out. Okay, then I get it.

N

The dashboard that is there, but we do not do much with it.

m

And why isn't it taken out? Is it because people can't get it out or does it take too much effort?

N

Well, then we come back to the first point, eh, that no one has been appointed within the hospital to maintain that dashboard and to implement care path improvements so that the ease of use is good. That things are adapted to new users, huh? To oncologists, to pathologists, et cetera. So it's not fine-tuned. Issues that we do have every now and then, such as the recent issue about the decline in the number of colorectal carcinomas. Whether that is due to the T1 carcinomas .. yes you can't figure that out.

m

Yes. Yes, exactly. Okay. So the data is there, but it is difficult to make it transparent.

N

Yes, the data is there, the infrastructure is there too. But you need people who are smart about it. And to answer the questions raised by specialists or other people involved in colon cancer. What's happening there?

m

Yes, okay, clear. I have now come to the final questions. If you look at the impact that improvement projects have had on clinical care. What kind of impact is that?

N

Difficult question.

m

Because you just indicated that that impact is actually little.

N

Yes, from the care path. But there might be other things in the field of surgery or something. I don't know where that much of an impact has been. That there has been a huge impact.

m

But in the things that you've been involved in, you haven't really had a very clear impact on the family on clinical care yet?

N

Well, maybe. We have. We now have all T1 carcinomas, we discuss on Thursday with the right people involved. So I think the decision-making process around T1 carcinomas has improved a lot.

m

Oh okay. But that is in any case nice to see that it really improves clinical care.

N

Yes, there are examples of that.

m

Yes, and when implementing such an improvement project, for example. Were there sometimes also unexpected effects, for example that the workload increased. Or the like?

N

Yes, I don't think so. I think that most improvement projects actually lead to an increase in job satisfaction and less work pressure.

m

Okay. And in your opinion are there certain factors that stimulate or hinder the implementation of an improvement project?

N

Well, what works against it is if more registration is required. Yes, people are very tired of that.

m

Yes, clearly. Last question, is there anything else you would like to share that you think is important for this interview?

N

No.

m

Okay.
